# Motivational valence alters memory formation without altering exploration of a real-life spatial environment

**DOI:** 10.1371/journal.pone.0193506

**Published:** 2018-03-20

**Authors:** Kimberly S. Chiew, Jordan Hashemi, Lee K. Gans, Laura Lerebours, Nathaniel J. Clement, Mai-Anh T. Vu, Guillermo Sapiro, Nicole E. Heller, R. Alison Adcock

**Affiliations:** 1 Center for Cognitive Neuroscience, Duke University, Durham, North Carolina, United States of America; 2 Department of Psychology, University of Denver, Denver, Colorado, United States of America; 3 Department of Electrical and Computer Engineering, Department of Biomedical Engineering, Department of Computer Sciences, Duke University, Durham, North Carolina, United States of America; 4 Department of Psychology and Neuroscience, Duke University, Durham, North Carolina, United States of America; 5 Department of Neurobiology, Duke University, Durham, North Carolina, United States of America; 6 Conservation Science, Peninsula Open Space Trust, Palo Alto, California, United States of America; 7 Department of Psychiatry and Behavioral Sciences, Duke University Medical Center, Durham, North Carolina, United States of America; University of Mississippi, UNITED STATES

## Abstract

Volitional exploration and learning are key to adaptive behavior, yet their characterization remains a complex problem for cognitive science. Exploration has been posited as a mechanism by which motivation promotes memory, but this relationship is not well-understood, in part because novel stimuli that motivate exploration also reliably elicit changes in neuromodulatory brain systems that directly alter memory formation, via effects on neural plasticity. To deconfound interrelationships between motivation, exploration, and memory formation we manipulated motivational state prior to entering a spatial context, measured exploratory responses to the context and novel stimuli within it, and then examined motivation and exploration as predictors of memory outcomes. To elicit spontaneous exploration, we used the physical space of an art exhibit with affectively rich content; we expected motivated exploration and memory to reflect multiple factors, including not only motivational valence, but also individual differences. Motivation was manipulated via an introductory statement framing exhibit themes in terms of Promotion- or Prevention-oriented goals. Participants explored the exhibit while being tracked by video. They returned 24 hours later for recall and spatial memory tests, followed by measures of motivation, personality, and relevant attitude variables. Promotion and Prevention condition participants did not differ in terms of group-level exploration time or memory metrics, suggesting similar motivation to explore under both framing contexts. However, exploratory behavior and memory outcomes were significantly more closely related under Promotion than Prevention, indicating that Prevention framing disrupted expected depth-of-encoding effects. Additionally, while trait measures predicted exploration similarly across framing conditions, traits interacted with motivational framing context and facial affect to predict memory outcomes. This novel characterization of motivated learning implies that dissociable behavioral and biological mechanisms, here varying as a function of valence, contribute to memory outcomes in complex, real-life environments.

## Introduction

Exploration can appear aimless, but it is not purposeless. In a world of limited resources, learning about the environment via open-ended exploration is crucial to an organism’s survival. Exploration enables discovery of new potential rewards and likely threats, and is centrally implicated in learning and memory. Yet despite its clear evolutionary necessity, open-ended exploration of a spatial environment is one aspect of motivated behavior that has received relatively little investigative attention. Moreover, the intuitive relationship between exploration and learning obscures a complicated causality; resolving this causality promises insights into both biological and behavioral bases of memory formation.

There can be little doubt that motivated exploration predicts enhanced memory. Experimental evidence has shown enhanced learning during volitional exploration [1–4], along with increased activation in the hippocampus and other medial temporal lobe substrates of memory encoding [5,6]. New research characterizing the neural architecture of human spatial memory and navigation has used virtual-reality mazes and city environments [7–11], characterized memory and its neural architecture in expert real-life navigators, [12,13], and, in a limited number of cases, contrasted navigation in real and virtual environments [14–16]. These studies are part of a rich literature in both animal and human models linking spatial memory and navigation to hippocampal function and episodic memory processes [7,17–21]. In all these instances, the motivated exploration of novel stimuli and environments is strongly associated with both hippocampal engagement and memory strength.

Despite these observations, the causality of relationships between exploration and memory remains ambiguous, because novel stimuli that motivate exploration also reliably elicit changes in neuromodulatory brain systems and directly alter memory formation, via effects on neural plasticity. For example, novelty that elicits exploration in experimental settings also elicits dopamine release. In addition to longstanding research implicating midbrain dopamine (DA) in a broad range of motivated and adaptive behaviors, including vigor [22,23], reward seeking, anticipation [24–27], and exploration in response to novelty [28–30], more recent work connects dopamine to enhanced memory formation. These memory enhancements are evident in response to both reward motivation [31–33] and novelty [34,35]. Duzel and colleagues [36] sought to synthesize these findings in a theoretical framework, NOMAD (*Novelty-related Motivation of Anticipation and exploration by Dopamine*), which posits that dopamine improves memory not only by enhancing plasticity and memory consolidation, but also by promoting increased activity and exploration in response to novel events.

Interestingly, however, novelty is not an unambiguous stimulus, and exploration of novelty can be modulated by affect and motivational states. Exploration of novel environments resembles behavioral responses to reward: both elicit approach, behavioral activation, and mesolimbic dopaminergic system activity [27,37]. Moreover, it has been proposed that, from an evolutionary perspective, novelty may hold inherent reward value [28,29]. However, novelty is not universally attractive or appetitive: for most organisms, exploratory responses to novelty only occur under conditions of expected reward and safety. Threat (for example, of electric shock) is robustly linked with reduced exploration [38,39], defensive freezing, and fleeing behaviors [40]. Thus, under threat, novelty may actually be aversive because of the uncertain potential for negative outcomes–i.e., “fear of the unknown” [41].

**Fig 1:**
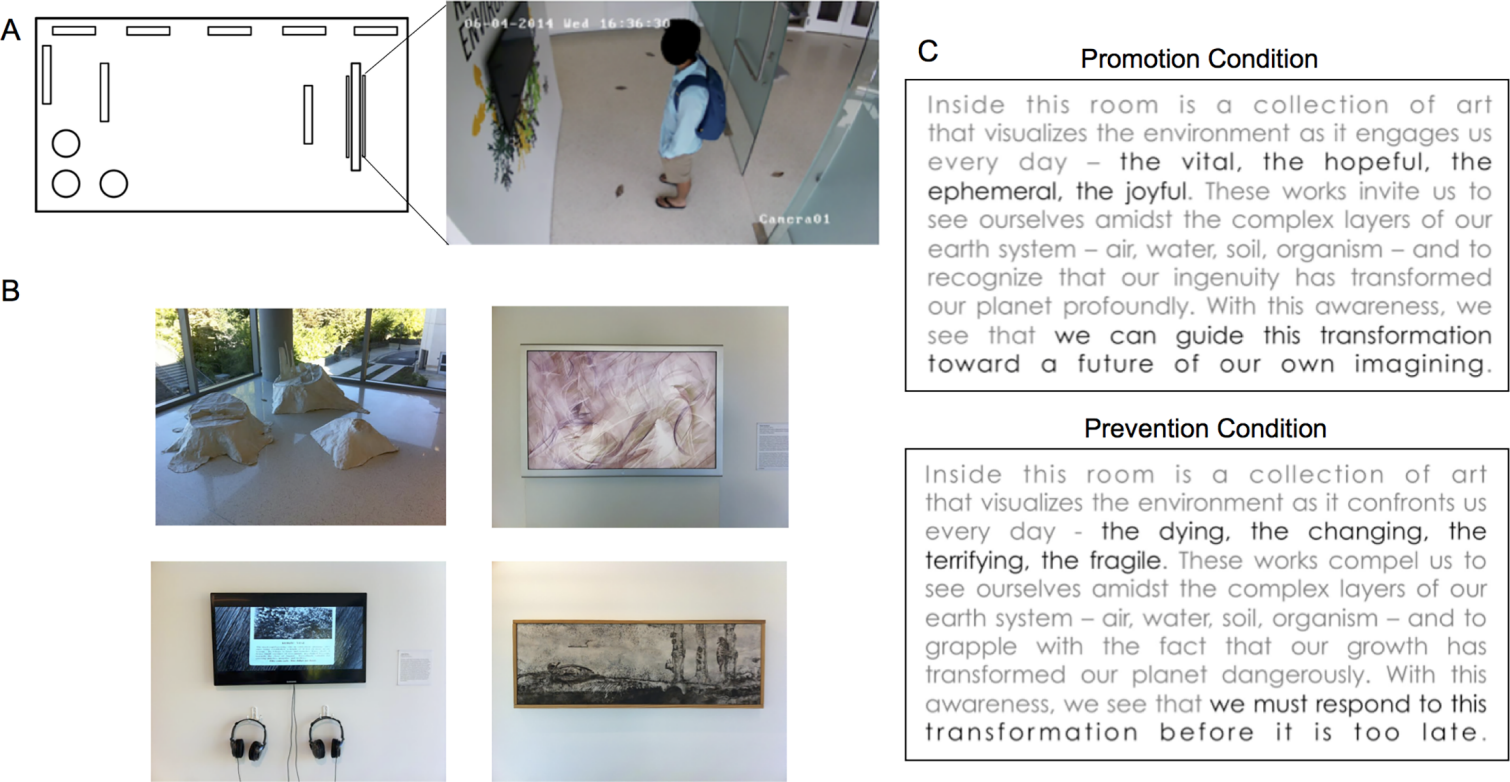
The art exhibit, Re-Imagining the environment. (a) Schematic of exhibit space (13.1m x 6.25m; ~82 square metres). A partial wall occluded the space at entry and displayed a monitor with the Promotion or Prevention-themed exhibit statement. (b) Examples of artwork in the exhibit, which explored the relationship between humans and the natural world. Eight pieces of art, of different media, were displayed. (c) Promotion and Prevention versions of the exhibit statement, where human response to environmental change was framed as pursuit of desired outcomes (Promotion), versus prevention of undesired outcomes (Prevention), to elicit distinct motivational states, as indexed by facial expressions of affect.

The multivalent nature of novelty creates an opportunity to deconfound effects of motivation and exploration on memory formation. To disambiguate these interrelationships, we manipulated motivational state prior to entering a spatial context, measured exploratory responses to that context and novel stimuli within it, and then examined motivation and exploration as predictors of memory outcomes. We conducted the study in a physical space–an art exhibit examining human relationships to the natural environment (entitled *Re-Imagining the Environment*, Fig 1). The gallery was equipped as an experimental space to elicit and quantify motivated exploration of space and multi-valenced art items. This setting permitted us replicate and extend our prior findings from a virtual spatial environment [11]. In addition, we used spatial and item memory measures sensitive to hippocampal and medial temporal lobe components of memory function. These measures allowed us to investigate for previously reported effects: namely, that affect [42,43] and motivational incentive valence [11,44] have specific, dissociable effects on memory performance and on the medial temporal lobe memory system [45].

Several additional aspects of our experimental design are of note. First, to avoid directly incentivizing exploration, we manipulated motivation by cueing goals for Promotion (i.e., advancement towards a rewarding outcome) or Prevention (avoidance of a punishing outcome) regulatory focus [46]. These cues appeared in written curatorial statements introducing the exhibit’s themes of environmental sustainability at the entrance of the exhibit. (Fig 1). Second, in this naturalistic setting with affectively rich stimuli, we expected cognition and behavior to show varied sources of influence, including individual differences reflecting dopaminergic genotype [47–50], trait regulatory focus of motivation [51,52], and attitudes about the themes of the exhibit (here, environmentalism; [53–55]). Moreover, our own prior work has demonstrated that moment-to-moment variability in mesolimbic DA circuit activity [31] and physiological arousal [11] predict motivated memory performance; thus, we used video analysis to classify facial expressions of affect while participants read the Promotion or Prevention cueing statements [56], allowing for temporally precise quantification of the impact of our motivational manipulation.

In sum, the current investigation aimed to disambiguate the relationships between motivational valence, exploratory behavior and memory, while accounting for momentary affect and potential interactions with individual differences in personality and attitudes. In accordance with accounts of dopamine-driven behavioral activation and exploration behaviors [27,36], we predicted that participants in the Promotion condition would explore the art exhibit more than those in the Prevention condition. Given evidence that reward motivation may specifically improve relational or context memory [11,44], while threat motivation and negative affect do not [11,42,43,57], we further predicted enhanced spatial memory in Promotion but no significant differences in item memory, as a function of framing condition. Finally, and, to our knowledge, uniquely in the extant literature, we sought to determine whether the influences of motivational valence on memory formation were attributable to, or independent of, changes in exploratory behavior.

## Methods

This study was approved by the Institutional Review Board at Duke University Medical Center (Protocol ID: Pro00053116).

### Participants

Ninety-eight participants were enrolled (51 female; mean age 32.9 +/- 1.5 years; range 18–71 years). Participants were recruited from the Duke University and Durham community using posted advertisements. Informed consent was obtained from all participants in accordance with human subjects guidelines established by the Institutional Review Board at Duke University Medical Center. Participants received institutionally standard compensation at the rate of approximately $10/hour, with no additional incentive for performance. Fifty-two participants took part in the Promotion condition and forty-six participants took part in the Prevention condition. Due to technical issues, certain portions of data were missing or unusable (usable N obtained for each data measure is noted in S1 Table in the Supporting Information). In particular, 16 participants did not have usable exploration video data, and a separate 15 participants did not have facial expression video data.

### Experiment procedure

**Fig 2:**
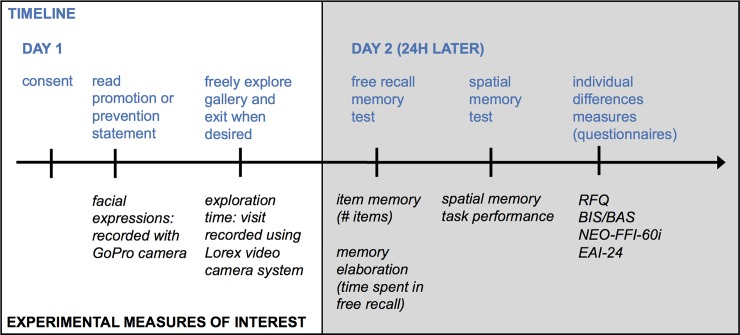
Experimental timeline and measures of interest collected. On Day 1, participants read either a Promotion or Prevention-oriented statement at entry and then freely explored the exhibit space, ending their visit at will. A wall-mounted GoPro camera recorded participants as they read the statement and an automated facial expression classifier was applied to the data to calculate participants’ angry, happy, sad, surprised, or neutral expressions as affective responses to the manipulation. A ceiling-mounted Lorex video system recorded participant activity through the exhibit: these data were used to calculate exploration time. Twenty-four hours later, participants provided open-ended free recall of their visit; this was audio-recorded. Participants next completed a spatial memory test of the exhibit, followed by individual difference measures.

The experiment exhibit is shown in Fig 1 and the experimental timeline is shown in Fig 2. The experiment took place on the campus of Duke University in two sessions occurring 24 hours apart. On Day 1, participants arrived at the laboratory and provided informed consent. Consent procedures indicated that compensation would occur after completion of the study on Day 2 at the rate of approximately $10/hour, with a full hour estimated for the gallery visit. No incentives were offered for better performance on Day 2. Following consent, participants were taken to the art exhibit in the experiment gallery (see *Art Exhibit*: *Re-Imagining the Environment*, below). All participants entered the gallery space alone, explored, and exited it at will. Prior to exhibit entry, participants were instructed to read in full the exhibit statement, presented on a flat screen monitor at entry (see *Motivational Framing Manipulation*, below) and to freely explore the exhibit, as prompted by the following experimenter script:

Through these glass doors is the art exhibit, “Re-Imagining the Environment”. Please read in full the opening statement, presented on a flat screen at the front of the exhibit, before continuing further into the exhibit. The opening statement is important to your experience.

Feel free to visit the art objects in the exhibit, as many as you like, in any order you choose, for as long as you wish. It is not necessary to check back in with me when you leave the exhibit, and I may not be here when you return.

Participants’ facial expressions were recorded as they read the exhibit statement at entry using a high-definition personal camera (GoPro Inc., San Mateo, CA) mounted above the statement display. Participant movement throughout the exhibit was recorded using a ceiling mounted security camera system (Lorex Technology, FLIR Systems, Wilsonville, OR). Upon the end of the self-paced visit, participants exited the exhibit without further interaction with the experimenter. Because our experimental design prioritized exhibit visit duration as a behavioral measure of interest, we elected not to conduct an immediate memory test following the exhibit visit, with the goal of minimizing perceived experimenter demand characteristics and enabling as naturalistic an exploration experience as possible.

Twenty-four hours following their exhibit visit, participants returned to the laboratory. Participants completed a verbal, digitally-recorded, free recall test of the exhibit in response to the following prompt, which was deliberately worded to encourage both recall of specific items as well as open-ended recall of contextual memory details:

The first thing we are interested in is your free recall of the exhibit. Please feel free to provide as much or as little detail about the exhibit and the objects as you wish. Please provide us with any of your impressions, details and emotions associated with the art. You have as much time as you want to complete this section. Please let me know when you are finished.

Following the free recall test, participants completed a self-paced, computerized spatial memory test of exhibit layout. Participants were presented with an onscreen rectangle representing the gallery space and icons symbolizing each of the individual art pieces. Using a computer mouse, participants were required to drag and drop each item to its appropriate spatial location in the gallery space and rate their memory confidence for each item on a 5-item Likert scale (1 = guessing; 5 = extremely confident). Finally, participants completed individual difference measures: the Behavioral Inhibition System/Behavioral Activation System (BIS/BAS) Scales [58], the 60-item NEO-Five Factor Inventory (NEO-FFI-60) [59], and the 24-item Environmental Attitudes Scale (EAI-24) [60]. BIS and BAS (an averaged composite of the BAS-Drive, BAS-Fun Seeking, and BAS-Reward Responsivity subscales), NEO-Neuroticism, NEO-Openness to Experience, and the EAI-Preservation subscale (used here as a general measure of environmental concern) were chosen as individual differences predictors of interest. These measures were chosen based on *a priori* associations with motivated behavior, exploration, and environmental engagement. However, because of the novelty of our paradigm, close experimental precedent was not available in prior literature; these predictors should thus be considered theoretically-motivated but exploratory.

### Art exhibit: *Re-Imagining the Environment*

The art exhibit (curated by N.E. Heller), entitled *Re-Imagining the Environment*, was located within a gallery space in Duke University’s Nicholas School of the Environment (Fig 1). The exhibit featured art in various media (painting, sculpture, video, printmaking, etc.) from nine contemporary American artists and, as an ensemble, was intended to explore the relationship between humans and the natural environment. Art was chosen specifically to vary in affective valence (i.e., exploring themes of environmental hope, despair, innovation, disgust, etc.). The gallery space was approximately 13.1m x 6.25m (82 square metres) and contained eight art objects.

### Motivational framing manipulation

Motivational context of the exhibit (Promotion vs. Prevention) was manipulated using a statement displayed on a freestanding wall at entry (occluding the rest of the exhibit) (Fig 1). The statement discussed the relationship between humans and the environment, as well as man-made environmental change, in terms of potential gain versus loss. Promotion and prevention versions of the statement were developed such that content was as closely matched as possible between them (Fig 1C).

Promotion version:

Inside this room is a collection of art that visualizes the environment as it engages us every day–**the vital, the hopeful, the ephemeral, the joyful.** These works invite us to see ourselves amidst the complex layers of our earth system–air, water, soil, organism–and to **recognize that our ingenuity** has transformed our planet **profoundly**. With this awareness, we see that **we can guide this transformation toward a future of our own imagining.**

Prevention version:

Inside this room is a collection of art that visualizes the environment as it confronts us every day–**the dying, the changing, the terrifying, the fragile.** These works invite us to see ourselves amidst the complex layers of our earth system–air, water, soil, organism–and to **grapple with the fact that our growth** has transformed our planet **dangerously**. With this awareness, we see that **we must respond to this transformation before it is too late.**

### Data analysis

#### Calculation of exploration and memory measures

Manual inspection of the Lorex video data was used to calculate total exhibit exploration time, as well as engagement times for individual art items, for each participant. Exploration time was calculated on the order of seconds. Total exploration time was calculated as the duration of time spent in the exhibit space from entry to exit, while item engagement time was calculated as the total duration of time spent engaging (visually and/or by touch) with the art item. Nine items were coded for item engagement time (the eight art objects in the exhibit, and the statement at entry). Additional explored items in the exhibit space (e.g., windows, flooring, emergency exit pull station) were not included in these calculations. From these measures, we also calculated an “item/wander time” measure: a proportion score of the total amount of time spent engaging with specific art items, divided by total time spent in the exhibit space. The higher this measure, the greater proportion of a participant’s total exhibit exploration time was spent engaging with art items (as opposed to “wandering” in the exhibit).

Multiple measures of memory performance were extracted from the free recall and spatial memory test data collected. Audio recordings of verbal free recall were transcribed and coded for item recall success (number of items recalled; calculated as an integer value of specific exhibit items mentioned) and item valence by two independent, condition-blind raters. Again, nine items (eight art objects and the entry statement) were included in these measurements. Item valence was coded as positive, negative, neutral/not-specified, or ambivalent. In addition to these measures of item memory, the time length of the free recall recording was taken as a measure of contextual memory for each participant (given the open-ended nature of our free recall prompt, which encouraged participants to generate contextual, elaborative memory details). Spatial memory performance was calculated from the spatial memory test, which was scored in terms of proportion accuracy (placement of each art item was scored as correct or incorrect) and memory confidence by item. Spatial memory was measured for the eight art items only, not the entry statement.

#### Analysis of exploration and memory measures

Between-subjects *t-*tests were used to examine whether total exploration time, item/wander time, item recall success, free recall time, or spatial memory accuracy significantly differed as a function of framing group. We also examined whether total exploration time or item/wander time significantly related to memory outcomes (item recall success, free recall time, and spatial memory accuracy) and whether these relationships differed with motivational framing condition using Pearson correlations, conducted separately in Promotion and Prevention condition groups. Finally, we tested for significant differences in recalled item valence (i.e., whether the proportions of art items that participants recalled as emotionally positive, negative, neutral, and ambivalent) significantly differed by groups. Given that the numbers of items recalled varied by individual, and valence proportions were non-independent, we used a mixed-effects linear logistical regression for this analysis. This approach also allowed us to examine item proportions within subjects and, by avoiding data aggregation, account for individual variability in the numbers of items recalled. The structure of this analysis is shown in Table 1, Row 1.

**Table 1:** Model structure for mixed-effects regressions used to examine relationships between motivational context, exploration, and memory outcomes.

	Row	Dependent Variable	Predictor Variables		Regression Model	R function and package used
			Fixed Effects	Random Effects		
**Summary-Level Analyses**	1	Recalled item valence (proportions)	Framing group	Subject Art item (nested within subject)	Linear	lme function in the nlme software package
	2	Facially expressed emotions during reading of framing statement (multinomial outcome)	Framing group	Subject Video frame (nested within subject)	Logistic	glmer function in the lme4 software package
**Item-Level Analyses**	3	Item engagement time (in seconds)	Framing group	Subject Art item (nested within subject)	Linear	lme function in the nlme software package
	4	Item recall success (binomial outcome)	Framing group Item engagement time	Subject Art item (nested within subject)	Logistic	glmer function in the lme4 software package
	5	Free recall time (in seconds)	Framing group Item engagement time	Subject Art item (nested within subject)	Linear	lme function in the nlme software package
	6	Recalled item valence (multinomial outcome)	Framing group Item engagement time	Subject Art item (nested within subject)	Logistic	glmer function in the lme4 software package
	7	Spatial memory accuracy (binomial outcome)	Framing group Item engagement time	Subject Art item (nested within subject)	Logistic	glmer function in the lme4 software package

At the summary level, this approach was used to examine whether Promotion vs. Prevention groups significantly differed in the emotional valences of recalled art items. At the item level, this approach was used to examine whether item engagement time differed as a function of group, as well as examining whether memory outcomes differed as a function of group, item engagement time, or the interaction between the two factors. All analyses were conducted in R software version 3.4.1 (www.r-project.org); function and software package is specified for each.

In addition to examining summary-level performance, we conducted analyses examining performance on the level of individual art items, with the goal of characterizing relationships between motivational context, exploration, and memory in our data on a more fine-grained level. Given that the numbers of art items explored and recalled varied on a subject-to-subject basis, we again used mixed-effects regression models for these analyses. We first examined the effect of motivational context on item engagement time, and then constructed four separate models to examine the effects of motivational context and item engagement time on item-level memory performance, with item recall success, free recall time, recalled item valence, and spatial memory accuracy as the dependent variables. The structure of these analyses is shown in Table 1, Rows 3–7.

Finally, exploration and memory measures were examined as a function of individual differences in affective response to the framing manipulation (as measured via facial expressions while reading the motivational framing statement at entry) and as a function of individual differences in personality and attitude measures. Analyses investigating these relationships are outlined below in *Categorization and Analysis of Facial Expressions* and *Examining Individual Differences in Personality and Attitude as Predictors of Exploration and Memory*.

#### Categorization and Analysis of Facial Expressions

GoPro video data of participants’ spontaneous emotional facial expressions while reading the motivational Promotion or Prevention-oriented exhibit statement were analyzed using an automated facial expression analysis algorithm proposed in [56]. These methods are described further in the Supporting Information (in *S1 Text: Supplementary Methods: Facial Expression Analysis*). The algorithm analyzed video of the face (collected at 30 frames/second at 1080p resolution and analyzed every 5 frames or 166.67ms) and classified the facial expression for each video frame as one of the following emotions: angry, happy, sad, surprised or neutral (or unclassifiable due to obscured view). From the classifiable data, we then examined whether proportions of video frames with a given expressed emotion differed by motivational framing condition (Promotion vs. Prevention) using a mixed-effects logistic regression (model structure summarized in Table 1, Row 2). Due to the very small amount of data classified as sad (<0.01%), this expression was eliminated from analysis, leaving angry, happy, surprised and neutral expressions to be compared across framing conditions.

Facial expressions were also examined as a predictor of subsequent memory performance. Prior work from our laboratory has demonstrated that individual variability in arousal interacted with motivational context to predict spatial memory, with arousal inversely predicting memory performance under reward but not penalty incentive [11]. We investigated whether similar relationships were present in the current dataset by correlating expressed surprise (a putative measure of arousal) with measures of subsequent exploration and memory, separately for Promotion and Prevention conditions.

#### Examining individual differences in personality and attitude as predictors of exploration and memory

To investigate relationships between trait individual differences, motivational context, exploration, and memory performance in the present paradigm, hierarchical multiple regression analyses were conducted with summary-level measures of exploration time, item/wander time, item recall success, free recall time, and spatial memory accuracy as dependent variables (DVs). Framing condition (Promotion/Prevention) and individual difference measures of interest (BIS, BAS, NEO-Openness to Experience, NEO-Neuroticism, and EAI-Preservation) were defined as predictors for these analyses.

These models were constructed with predictor variables entered in two steps. In the first step of the regression mode for each DV, our individual difference measures of interest (BIS, BAS, NEO-Neuroticism, NEO-Openness, and EAI-Preservation) and framing condition were entered as predictors. To test whether individual differences interacted with framing condition to predict behavior, interaction terms with framing (collectively referred to as “Indiv x Framing” terms) were entered for each predictor in a second step. These interaction terms were entered in a second step to test for their predictive ability above and beyond the main effects of individual differences and framing condition. For the memory analyses, exploration time was also added as a second-step predictor (again, to control for Step 1 effects).

## Results

Results are organized to address the three levels of relationships among motivational state, exploration, and memory: 1) group-level analyses of affect, exploration and memory for the entire exhibit, 2) analyses on the individual item level; 3) analyses of how the motivational framing manipulations interacted with individual beliefs and temperament, including facial expressions of affect during motivational statement reading, to predict exploration and memory.

### Framing manipulation effects on affect, motivated exhibit exploration, and memory

#### Did affective facial responses while reading framing statement differ with motivation condition?

**Fig 3:**
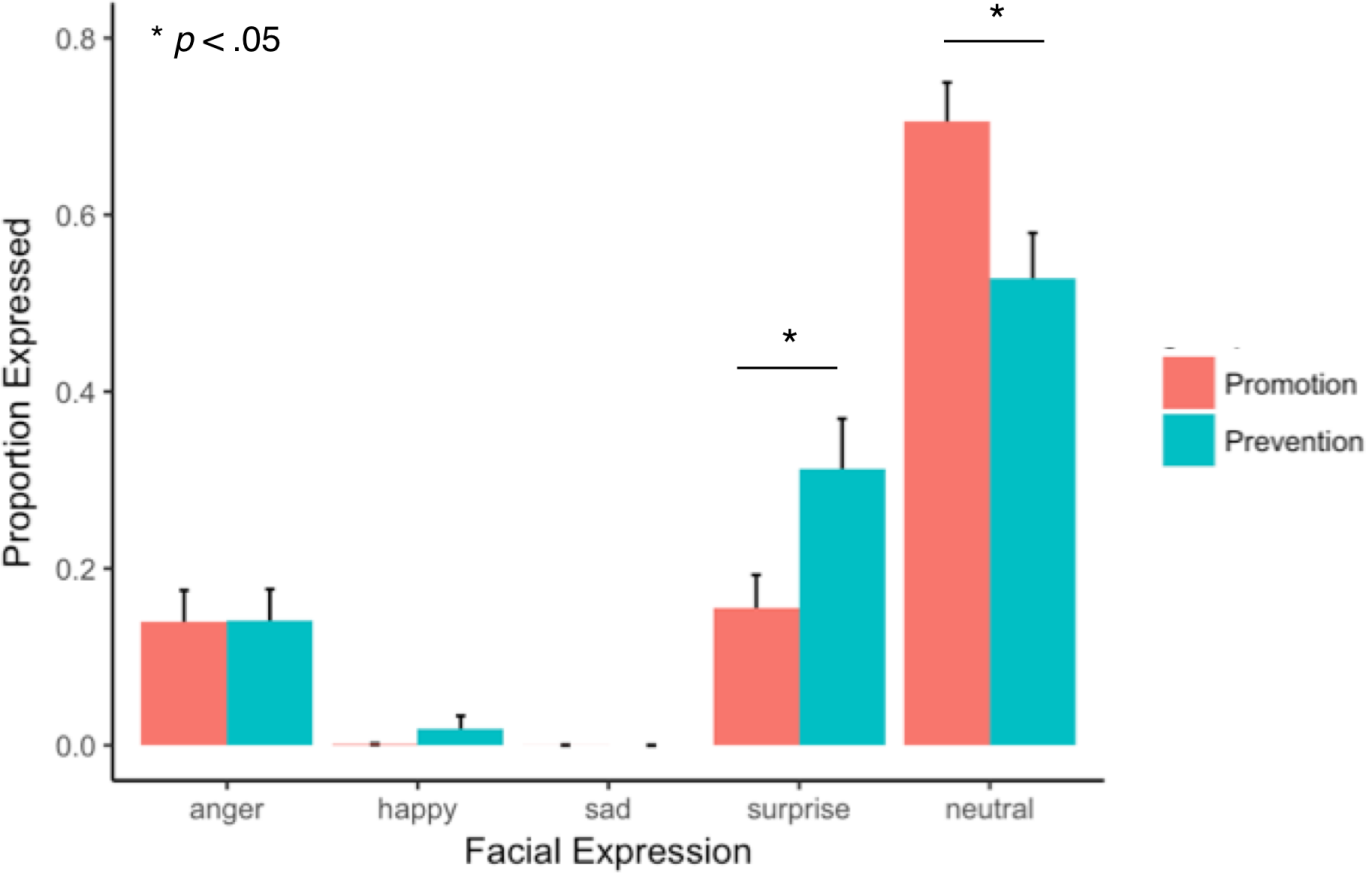
Facial expressions while reading motivational statement as a function of Promotion vs. Prevention framing. Participants expressed significantly more surprise (and correspondingly, less neutral expression) in Prevention vs. Promotion. These findings confirm that participants had differing affective responses to the Promotion and Prevention-oriented versions of the exhibit: specifically, participants expressed more surprise in response to the statement in the Prevention condition.

On average, participants in each condition viewed the cue statement for ~30 seconds (Promotion *M*(43) = 30.97 seconds, *SD* = 13.41; Prevention *M*(39) = 30.77 seconds, *SD* = 13.94); viewing time did not significantly differ between conditions [*t*(81) = -.081, *p* = .986, Cohen’s *d* = .015]. Video data of participants’ facial expressions (N = 83) during statement reading was automatically classified as angry, sad, surprised, neutral, happy, or unclassifiable (see Methods: *Data Analysis*: *Categorization and Analysis of Facial Expressions*); 20.2% of the data was unclassifiable due to obscured view. Of the classifiable data, across Promotion/Prevention conditions, faces were classified most as neutral (61.1%), then surprised (23.8%) and angry (14.2%), with very few frames classified as happy (0.9%) or sad (<0.01%) (shown in Fig 3 separately for each framing condition). Mixed-effects logistic regression (described in *Methods)* revealed that the effect of framing condition was significant for the contrast of surprise vs. neutral expressions [β = -1.3872, SE = 0.5561, z = -2.495, p = .0126], with greater neutral in Promotion vs. Prevention, and greater surprise in Prevention vs. Promotion. No other contrasts reached significance.

#### Did exploration and memory of the exhibit differ in promotion vs. prevention condition?

As measures of exploration, we calculated total exhibit visit time from video data. We also calculated “item/wander time” the proportion of total exploration time that was spent engaging with art items (vs. “wandering”). As measures of memory, we calculated item recall success (number of items recalled), valence of items recalled (emotionally positive, negative, neutral, or ambivalent), free recall time, and spatial memory accuracy.

Contrary to the hypothesis that both exploration and spatial memory would be enhanced in the Promotion condition relative to Prevention, no measure of exploration or memory significantly differed as a main effect of framing condition. Summary measures of valence of remembered items (proportion of items recalled as emotionally positive, negative, neutral, or ambivalent) also did not significantly differ as a function of framing condition. Statistics are presented in Table 2; full analyses are provided in the Supporting Information (*S2 Text: Supplementary Results. Exploration and Memory Measures as a Main Effect of Group*). Thus, overall motivation to remain in the gallery and overall memory appeared to be equivalent between groups.

**Table 2:** Exploration time, number of items recalled, item valence in free recall, free recall time, and spatial memory performance, separated by framing condition.

		Promotion (N = 52)		Prevention (N = 46)		Group Difference Test		
		usable n	mean (SD)	usable n	mean (SD)	t-statistic	p-value	Cohen’s d
**Exploration time (seconds)**		49	1185.6 (796.9)	46	1204.5 (824.2)	-0.114	0.909	-0.23
**Item/wander time (proportion of total exploration time spent in item engagement)**		43	0.835 (0.087)	39	0.824 (0.139)	0.474	0.637	0.104
**Number of items recalled**		50	6.38 (2.49)	41	5.95 (3.04)	0.740	0.461	0.156
**Item valence in free recall (percentages)**	**Positive**	50	56.2 (27.5)	38	48.7 (29.9)	1.218	0.226	0.261
	**Negative**	50	11.0 (16.7)	38	13.2 (14.5)	-0.643	0.522	-0.140
	**Neutral**	50	30.5 (29.4)	38	35.5 (34.9)	-0.726	0.470	-0.154
	**Ambivalent**	50	2.6 (7.5)	38	4.5 (11.3)	-0.908	0.367	-0.190
**Time in free recall (seconds)**		50	261.62 (231.75)	41	265.63 (191.93)	-0.089	0.930	0.019
**Spatial memory performance (proportion accuracy)**		50	0.770 (0.278)	43	0.799 (0.311)	-0.481	0.631	0.100
**Spatial memory confidence (5-point Likert scale, from 1 = “guessing” to 5 =“very confident”)**		50	4.12 (0.951)	43	4.20 (1.05)	-0.381	0.704	-0.079

#### Did total exploration time predict memory outcomes?

**Fig 4:**
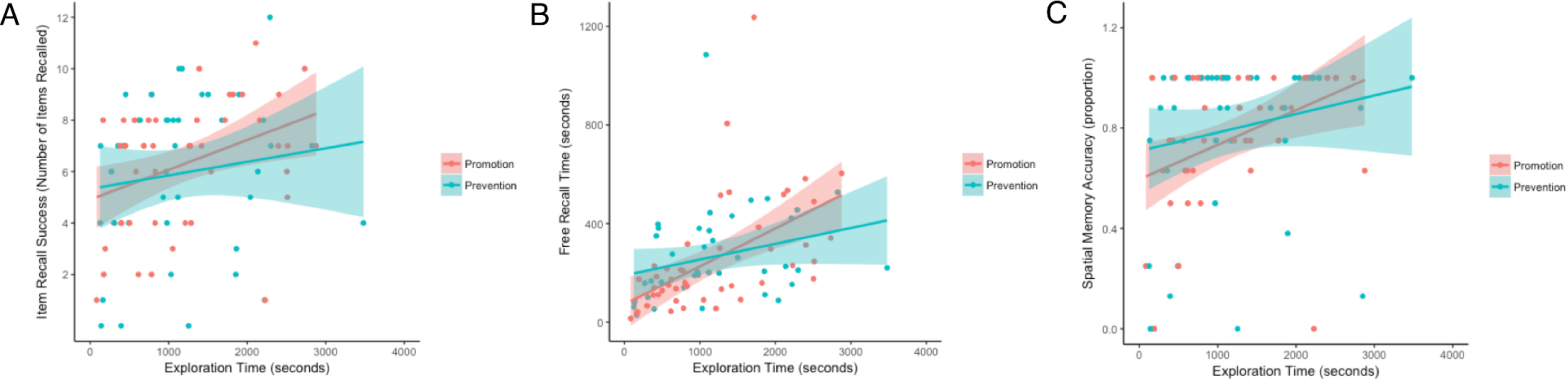
Relationships between exploration and memory measures as a function of motivational framing. Exploration time was positively associated with (a) item recall success, (b) free recall time, and (c) spatial memory accuracy; however, these relationships were statistically significant only in the Promotion condition, and not significant in the Prevention condition. Line shading indicates standard error.

A critical question for the current study was whether we would observe relationships between exploration time and memory that could account for motivational influences on memory. Separate Pearson correlations for Promotion and Prevention conditions revealed that in the Promotion condition, exploration time was significantly associated with all three memory outcomes [item recall success: *r*(47) = .371, *p* = .010; free recall time: *r*(47) = .535, *p* < .001; spatial memory: *r*(47) = .391, *p* = .007]; correlations of exploration time with free recall time and spatial location memory survived Bonferroni correction. In contrast, in the Prevention condition, exploration time did not significantly correlate with any memory outcomes [item recall success: *r*(41) = .140, *p* = .383; free recall time: *r*(41) = .270, *p* = .088; spatial memory accuracy: *r*(41) = .195, *p* = .209]. Correlation strengths did not significantly differ by condition, however [item recall success: *z* = 1.12, *p* = .263; free recall time: *z* = 1.45, *p* = .147; spatial memory: *z* = 0.97, *p* = .332; all comparisons two-tailed]. These relationships are shown in Fig 4.

### Item-level analyses of exploration and memory

#### Did exploration and memory of individual items differ in Promotion vs. Prevention condition?

To investigate exploration-memory relationships on the level of individual art items, mixed-effects linear regression analyses were used to construct five models. Item engagement time, item recall success, free recall time, recalled item valence, and spatial memory accuracy were defined as outcome variables.

Item engagement time. With framing group defined as a fixed effect and subject and art item as random effects, framing group was not a significant predictor of item engagement time (β = -8.3779, SE = 18.7906, t = -0.4459, p = .6571).

Item recall success. With item engagement time, framing group, and the interaction between the two defined as fixed effects and subject and art item as random effects in a mixed-effects linear logistical regression, item engagement time significantly predicted item recall success, with longer engagement times associated with successful recall (β = 0.0125, SE = 0.0031, z = 4.023, p < .001). While framing group as a main effect was not a significant predictor of recall (β = -0.4012, SE = 0.3537, z = -1.134, p = .257), the interaction of item engagement time and framing group was significant (β = -0.0095, SE = 0.0033, z = -2.831, p = .005), with the predictive relationship between item engagement time and successful recall was significantly stronger in Promotion versus Prevention.

Free recall time. With item engagement time, framing group, and the interaction between the two defined as fixed effects and subject and art item as random effects, item engagement time significantly predicted free recall time, with longer item engagement time positively associated with longer free recall time (β = 0.0630, SE = 0.0070, t = 8.974, p < .001). Framing group as a main effect was not a significant predictor of free recall time (β = 2.7252, SE = 6.0026, t = 0.454, p = .6513). However, the interaction of item engagement time and framing group was significant (β = -0.0372, SE = 0.0113, z = -3.294, p = .001), indicating that the predictive relationship between item engagement time and free recall time was significantly stronger in Promotion versus Prevention.

Recalled item valence. Analyses of whether an item was recalled as emotionally positive, negative, neutral, or ambivalent (coded by two independent raters) were conducted as binomial contrasts between outcome categories (following [61]): with four valence outcomes, six separate binomial contrasts were computed. The contrast of positive vs. neutral revealed a significant effect of item engagement time, with longer item engagement times associated with recall of items as more positive (β = 0.0120, SE = 0.0035, z = 3.450, p < .001); this effect was further qualified by a significant interaction between item engagement time and framing group (β = -0.0105, SE = 0.0036, z = -2.920, p = .004), indicating that the relationship between item engagement time and subsequent positive item recall was again more robust in the Promotion vs. Prevention group. The contrast of negative vs. neutral memory recall also revealed a significant effect of item engagement time, with longer engagement time with recall of items as neutral rather than negative (β = 0.0057, SE = 0.0029, z = 1.992, p = .0464). A trend-level interaction between item engagement time and framing group (β = -0.0060, SE = 0.0034, z = -1.770, p = .0768), indicated that this relationship was again more robust in Promotion vs. Prevention. No other significant predictors were observed.

Spatial memory accuracy. With item engagement time, framing group, and the interaction between the two defined as fixed effects and subject and art item as random effects, none of the fixed effects significantly predicted spatial memory accuracy (item engagement time: β = 0.0009, SE = 0.0010, t = 0.853, p = .394; framing group: β = 0.9063, SE = 1.4204, t = 0.638, p = .5234; item engagement time × framing group interaction: β = -0.0001, SE = 0.0012, t = -0.114, p = .909).

In sum, the results of these item-level analyses are similar to results for summary-level exhibit exploration and memory measures: item engagement was positively correlated with item but not spatial memory outcomes, and more strongly correlated in the Promotion than Prevention condition. Longer item engagement times were also associated with the tendency to recall the art items more positively. All of these encoding-memory relationships were significantly stronger in Promotion than Prevention.

### Effects and interactions of individual differences

#### Did affective facial expressions while reading framing statement predict subsequent behavior?

**Fig 5:**
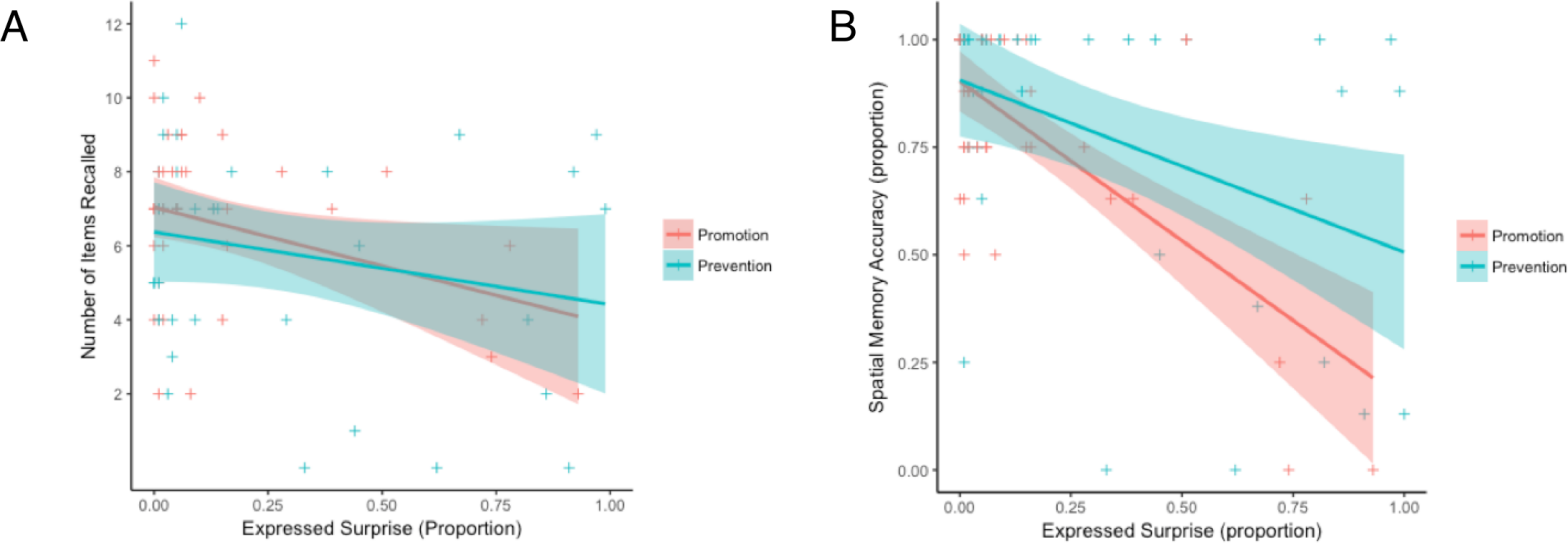
Item recall success and spatial memory accuracy as a function of expressed surprise and motivational framing. Expressed surprise (a) negatively predicted subsequent item recall success in Promotion framing (n.s. in Prevention framing); and (b) negatively predicted subsequent spatial memory accuracy in both framing conditions. This relationship was significantly stronger in Promotion framing. Line shading indicates standard error.

Taking expressed surprise as a putative measure of arousal, we measured the proportion of video frames during statement reading where participants’ facial expressions of affect were identified as surprise. We conducted Pearson correlations between surprise and behavioral measures (total exploration time, item recall success, free recall time, spatial memory accuracy), separately for Promotion and Prevention. Surprise and item recall success (shown in Fig 5A) were significantly negatively correlated in Promotion [*r*(40) = -.340, *p* = .032] but not Prevention [*r*(35) = -.219, *p* = .206]; however, these correlations did not significantly differ in strength (*z* = -0.54, *p* = .589, two-tailed). Surprise and spatial memory accuracy (shown in Fig 5B) were significantly negatively correlated in both Promotion [*r*(41) = -.708, *p* < .001] and Prevention [*r*(40) = -.374, *p* = .017]; this correlation was significantly stronger in the Promotion group (z = -.212, *p* = .034, two-tailed). Finally, the correlation of surprise with spatial memory accuracy was significantly stronger than with item recall success (z = -.212, *p* = .022, two-tailed). While these correlations of surprise with behavioral measures should be considered exploratory, the negative correlation between surprise and spatial memory in the Promotion condition was robust, surviving Bonferroni correction for multiple comparisons. In sum, in the Promotion condition, the greater the surprise (i.e., arousal) elicited by the motivation manipulation, the poorer subsequent memory was, particularly spatial memory.

#### Do trait individual differences predict exploration and memory behavior?

Because our paradigm used affectively rich stimuli and a complex environment to elicit spontaneous exploration, we expected greater behavioral variability and included measures of individual differences in personality and attitudes to help account for this variability, alone or in interaction with framing condition. We used hierarchical multiple regression analyses with predictors entered in two steps–framing condition and individual differences in Step 1, and Indiv x Framing interaction terms (and, for analyses with memory outcomes, exploration time) in Step 2 (as described above in *Methods*). In addition to these regression analyses, we also carried out Pearson correlations between each trait individual difference measure collected and our behavioral outcomes (exploration time, item/wander time, number of items recalled, free recall time, and spatial memory accuracy). These analyses are shown in the Supporting Information, S3 Table.

**Fig 6:**
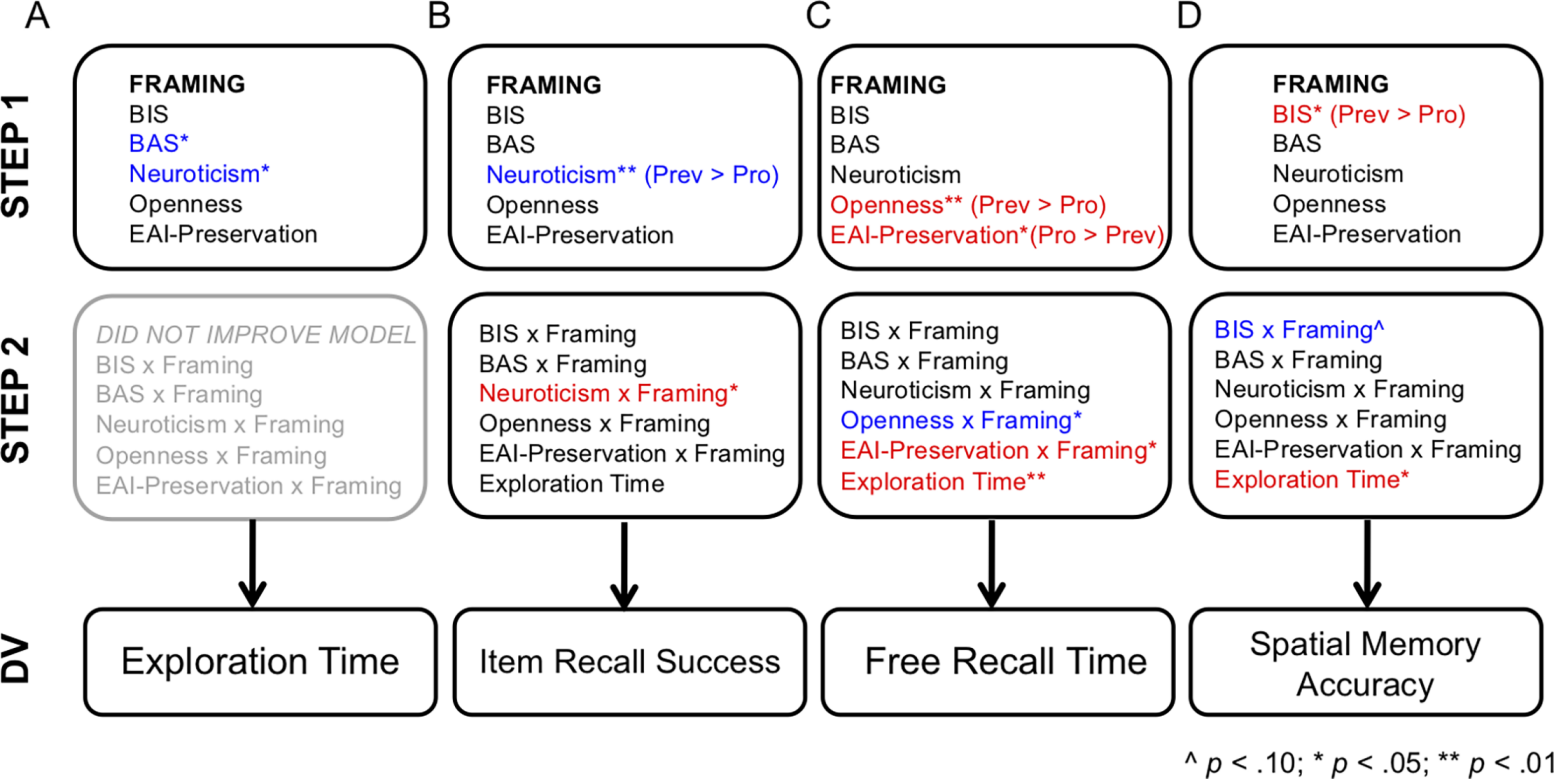
Hierarchical multiple regression analyses conducted to examine effects of individual differences predictors on exploration and memory dependent variables (DVs). Analyses are shown for the following DVs: (a) exploration time (only interpreted to Step 1); (b) item recall success (number of items recalled); (c) free recall time; (d) spatial memory accuracy. Predictors are indicated as being entered at Step 1 or Step 2, and statistically significant and trend-level individual predictors are indicated using superscripts (^*p* < .10; *p < .05; **p < .01). For predictors that significantly interacted with framing condition, the direction of the interaction is indicated (i.e., whether the predictor-DV relationship was stronger in Promotion [Pro] or Prevention [Prev]). Red or blue text coloring indicates whether the beta coefficients of significant and trend-level individual predictors were positive (red) or negative (blue).

Four regression analyses are presented here, with summary measures of exploration time, item memory (number of items recalled), free recall time, and spatial memory accuracy as dependent variables (DVs). A schematic of the model structure for each analysis, indicating significant predictors for each DV, is shown in Fig 6. Significant effects for each analysis are described below (with full statistics presented in Tables 2–5).

**Table 3:** Hierarchical multiple regression model with exploration time as a DV and individual differences and framing condition as predictors (Indiv x Framing interaction terms entered at Step 2).

**MODEL 1** **(R**^**2**^ **= .152, F(6,77) = 2.301, p = .043)**			
**Variable**	**β Coefficient**	**t value**	**p value**
Promotion/Prevention Framing	-.034	-.309	.758
BAS (Composite)*	-.239*	-2.208*	.030*
BIS	.015	.126	.900
NEO Neuroticism*	-.325*	-2.525*	.014*
NEO Openness to Experience	.196	1.632	.107
EAI Preservation	.045	.402	.689
**MODEL 2** **(R**^**2**^ **= .244, F(11,72) = 2.112, p = .030)**			
**Variable**	**β Coefficient**	**t value**	**p value**
Promotion/Prevention Framing	-.069	-.625	.534
BAS (Composite)	-.165	-1.090	.279
BIS	.221	1.228	.224
NEO Neuroticism*	-.405*	-2.013*	.048*
NEO Openness to Experience	.038	.205	.838
EAI Preservation	-.273	-1.393	.168
BAS (Composite) x Framing	-.075	-.514	.609
BIS x Framing	-.188	-1.118	.267
NEO Neuroticism x Framing	.080	.423	.674
NEO Openness to Experience x Framing	.235	1.369	.175
EAI Preservation x Framing*	.378*	2.051*	.044*

^*p* < .10

*p < .05

**p < .01

**Table 4:** Hierarchical multiple regression model with item memory (number of items recalled) as a DV and individual differences, framing condition, and exploration time as predictors (Indiv x Framing interaction terms and exploration time entered at Step 2).

**MODEL 1** **(R**^**2**^ **= .142, F(6,72) = 1.994, p = .078)**			
**Variable**	**β Coefficient**	**t value**	**p value**
Promotion/Prevention Framing	.120	1.048	.298
BAS (Composite)	.016	.141	.888
BIS	.095	.792	.431
NEO Neuroticism^	-.240^	-1.855^	.068^
NEO Openness to Experience*	.259*	2.114*	.038*
EAI Preservation	.155	1.337	.185
**MODEL 2** **(R**^**2**^ **= .269, F(12,66) = 2.022, p = .036)**			
**Variable**	**β Coefficient**	**t value**	**p value**
Promotion/Prevention Framing	-.126	1.127	.264
BAS (Composite)	.021	.141	.888
BIS	.146	.793	.430
NEO Neuroticism**	-.579	-2.879	.005
NEO Openness to Experience	.274	1.428	.158
EAI Preservation	.006	.028	.978
Exploration Time	.175	1.455	.150
BAS (Composite) x Framing	.031	.206	.837
BIS x Framing	-.055	-.309	.758
NEO Neuroticism x Framing*	.481*	2.543*	.013*
NEO Openness to Experience x Framing	-.020	-.111	.912
EAI Preservation x Framing	.154	.744	.460

^*p* < .10

*p < .05

**p < .01

**Table 5:** Hierarchical multiple regression model with free recall time as a DV and individual differences, framing condition, and exploration time as predictors (Indiv x Framing interaction terms and exploration time entered at Step 2).

**MODEL 1** **(R**^**2**^ **= .167, F(6,72) = 2.404, p = .036)**			
**Variable**	**β Coefficient**	**t value**	**p value**
Promotion/Prevention Framing	.016	.139	.890
BAS (Composite)	-.127	-1.155	.252
BIS	.094	.792	.431
NEO Neuroticism*	-.261*	-2.044*	.045*
NEO Openness to Experience*	.245*	2.027*	.046*
EAI Preservation^	.221^	1.929^	.058^
**MODEL 2** **(R**^**2**^ **= .334, F(12,66) = 2.753, p = .004)**			
**Variable**	**β Coefficient**	**t value**	**p value**
Promotion/Prevention Framing	-.012	-.110	.913
BAS (Composite)	-.050	-.350	.727
BIS	.065	.370	.713
NEO Neuroticism^	-.348^	-1.811^	.075^
NEO Openness to Experience**	.496**	2.708**	.009**
EAI Preservation	-.184	-.918	.362
Exploration Time**	.316**	2.749**	.008**
BAS (Composite) x Framing	-.029	-.202	.841
BIS x Framing	.017	.101	.920
NEO Neuroticism x Framing	.172	.952	.345
NEO Openness to Experience x Framing*	-.348*	-2.008*	.049*
EAI Preservation x Framing*	.420*	2.130*	.037*

^*p* < .10

*p < .05

**p < .01

(We also wish to note that a similar regression analysis examining the effect of trait individual differences, with item/wander time as the dependent variable, was also conducted. However, neither overall model fit, nor any individual differences as predictors, were observed to reach statistical significance. This may reflect the fact that, on average, participants spent most of their total exploration time in active item engagement and behavioral variability in item/wander time was relatively low. Full results for this analysis are in S2 Text and S4 Table.)

Exploration time. N = 84 (45 Promotion, 39 Prevention) were usable in this analysis (shown in Fig 6A and Table 3). The model reached significance at Step 1 [*F*(6,77) = 2.301, *p* = .043, R^2^ = .152]. Neuroticism and BAS were both significant negative predictors of exploration time [Neuroticism: β = -.325, part *r* = -.265, *p* = .014; BAS: β = -.239, part *r* = -.232, *p* = .030]; i.e., individuals higher in Neuroticism or BAS explored the exhibit for shorter amounts of time. With Step 2, R^2^ increase from Step 2 was not significant (ΔR^2^ = .092, Δ*F* = 1.751, *p* = .134). Thus, Step 2 was not interpreted further. This analysis indicates that individual differences (specifically, BAS and Neuroticism) predicted exploration time, but that inclusion of Indiv x Framing interaction terms did not significantly improve the model. Thus, effects of individual differences on exploration time manifested similarly across both framing conditions.

Item recall success. N = 79 (43 Promotion, 36 Prevention) were usable in this analysis (shown in Fig 6B and Table 4). The model reached trend-level significance at Step 1 [*F*(6,72) = 1.994, *p* = .078, R^2^ = .142]; the only significant predictor at this step was Openness, which positively predicted memory (β = .259, part *r* = -.231, *p* = .038). With addition of exploration time and interactions in Step 2, the model reached significance [*F*(12,66) = 2.022, *p* = .036, R^2^ = .269]; the R^2^ increase from Step 1 to Step 2 was marginally significant (ΔR^2^ = .136, Δ*F* = 1.901, *p* = .094). In this model, a significant Neuroticism x Framing interaction (β = .481, part *r* = .268, *p* = .013) indicated that Neuroticism negatively predicted item recall success in the Prevention condition (simple main effect: β = -.579, part *r* = -.303, *p* = .005) but not in the Promotion condition (simple main effect: β = -.074, part *r* = .047, *p* = .658). Taken together, these results suggest that individual differences, in interaction with motivational context, influence item memory. Notably, total exploration time was not a significant predictor of item recall success in this analysis.

Free recall time. N = 79 (43 Promotion, 36 Prevention) were usable in this analysis (shown in Fig 6C and Table 5). The model was significant at Step 1 [*F*(6,72) = 2.404, *p* = .036, R^2^ = .167]: Openness was a significant positive predictor (β = .245, part *r* = .218, *p* = .046), Neuroticism was a significant negative predictor (β = -.261, part *r* = .220, *p* = .045), and EAI-Preservation was a trend-level positive predictor (β = .221, part *r* = .207, *p* = .058). With Step 2, the model remained significant [*F*(12,66) = 2.753, *p* = .004, R^2^ = .334]. Further, the increase in R^2^ from Step 1 was significant (ΔR^2^ = .212, Δ*F* = 2.750, *p* = .019), indicating a significantly improved model fit with the addition of exploration time and interactions in Step 2. With Step 2, a significant EAI-Preservation x Framing interaction was present (β = .420, part *r* = .214, *p* = .037), indicating that EAI-Preservation significantly predicted free recall time in the Promotion condition (simple main effect: β = .333, part *r* = .255, *p* = .014), but not the Prevention condition (simple main effect: β = -.184, part *r* = -.092, *p* = .362). A significant Openness x Framing interaction was also present (β = -.348, part *r* = -.202, *p* = .049), indicating that Openness predicted free recall time in Prevention (simple main effect: β = .496, part *r* = .272, *p* = .009) but not Promotion (simple main effect: β = .018, part *r* = .012, *p* = .907). Exploration Time was also a significant positive predictor of free recall time (β = .316, part *r* = .276, *p* = .008).

These results indicate that individual difference measures, including exploration time, significantly predicted free recall time. Further, EAI-Preservation and Openness interacted with framing condition: EAI-Preservation was a stronger predictor of recall time in the Promotion condition, while Openness was a stronger predictor of recall time in the Prevention condition.

Spatial memory accuracy. N = 80 (43 Promotion, 37 Prevention) were usable for this analysis (shown in Fig 6D and Table 6). Step 1 of the model was significant [*F*(6,73) = 2.840, *p* = .015, R^2^ = .189]; model fit was driven by BIS (β = .260, part *r* = .230, *p* = .032) and EAI-Preservation (β = .261, part *r* = .239, *p* = .026), both of which were significant positive predictors of spatial memory performance. With addition of exploration time and interactions in Step 2, the model remained significant [*F*(12,67) = 2.487, *p* = .009, R^2^ = .308]; R^2^ increase from Step 1 to Step 2 was marginally significant (ΔR^2^ = .119, Δ*F* = 1.920, *p* = .090). At Step 2, a trend-level BIS x Framing interaction (β = -1.301, part *r* = -.173, *p* = .094) suggested that BIS predicted spatial memory in the Prevention condition (simple main effect: β = .450, part *r* = .258, *p* = .013), but not in the Promotion condition (simple main effect: β = .042, part *r* = .026, *p* = .798). Exploration Time also significantly predicted spatial memory (β = .276, part *r* = .239, *p* = .021).

**Table 6:** Hierarchical multiple regression model with spatial memory as a DV and individual differences, framing condition, and exploration time as predictors (Indiv x Framing interaction terms and exploration time entered at second step).

**MODEL 1** **(R**^**2**^ **= .189, F(6,73) = 2.840, p = .015)**			
**Variable**	**β Coefficient**	**t value**	**p value**
Promotion/Prevention Framing	-.083	-.745	.459
BAS (Composite)	-.093	-.840	.404
BIS*	.260*	2.186*	.032*
NEO Neuroticism	-.154	-1.203	.233
NEO Openness to Experience	.195	1.626	.108
EAI Preservation*	.261*	2.272*	.026*
**MODEL 2** **(R**^**2**^ **= .308, F(12,67) = 2.487, p = .009)**			
**Variable**	**β Coefficient**	**t value**	**p value**
Promotion/Prevention Framing	-.069	-.621	.537
BAS (Composite)	.052	.344	.732
BIS*	.450*	2.538*	.013*
NEO Neuroticism	-.181	-.892	.376
NEO Openness to Experience	.145	.792	.431
EAI Preservation	.193	.954	.344
Exploration Time*	.276*	2.356*	.021*
BAS (Composite) x Framing	-.139	-.958	.341
BIS x Framing^	-.273^	-1.700^	.094^
NEO Neuroticism x Framing	.118	.638	.526
NEO Openness to Experience x Framing	-.030	-.178	.859
EAI Preservation x Framing	.030	.153	.879

^*p* < .10

*p < .05

**p < .01

These results indicate that individual differences (including exploration time) predicted spatial memory; tentative evidence further suggested that BIS interacted with motivational framing to predict spatial memory more accurately under Prevention than Promotion context.

To summarize, individual differences analyses indicate that for free recall time and spatial memory accuracy, individual differences (including variation in exploration time) improved predictions over Framing condition. Contrasting with relationships between encoding behavior and memory success, where more robust relationships were seen only under Promotion versus Prevention and surprise, some trait individual difference measures were stronger predictors of memory under Prevention and some under Promotion. Sources and interpretations of these trait effects are discussed below.

## Discussion

Exploration is a plausible potential mechanism by which motivation can influence memory, but laboratory paradigms have been limited in their abilities to elicit and characterize exploratory behavior. Additionally, both exploration and memory formation may differ as a function of motivational context, in association with differential underlying neural circuitry. Reward or approach motivation has been observed to enhance dopaminergic midbrain activity and promote midbrain connectivity to hippocampus, enhancing exploratory behaviors [27,36] and contextual memory [31,33]. In contrast, threat or avoidance motivation, has been observed to promote amygdala activity and connectivity to cortical medial temporal lobe regions [62], and to reduce exploratory behavior [38,39], enhancing item but not contextual memory, similar to patterns seen under negative affect [42,43]. The present study compared profiles of volitional exploratory behavior under promotion and prevention motivation in a complex, real-life spatial environment, employing multiple memory measures characterizing both item and relational memory, to examine exploratory encoding behavior as a potential mechanism for motivated memory. Further, we explicitly examined the role of individual difference measures and their potential interactions with motivational context to predict encoding behavior and memory outcomes.

**Fig 7:**
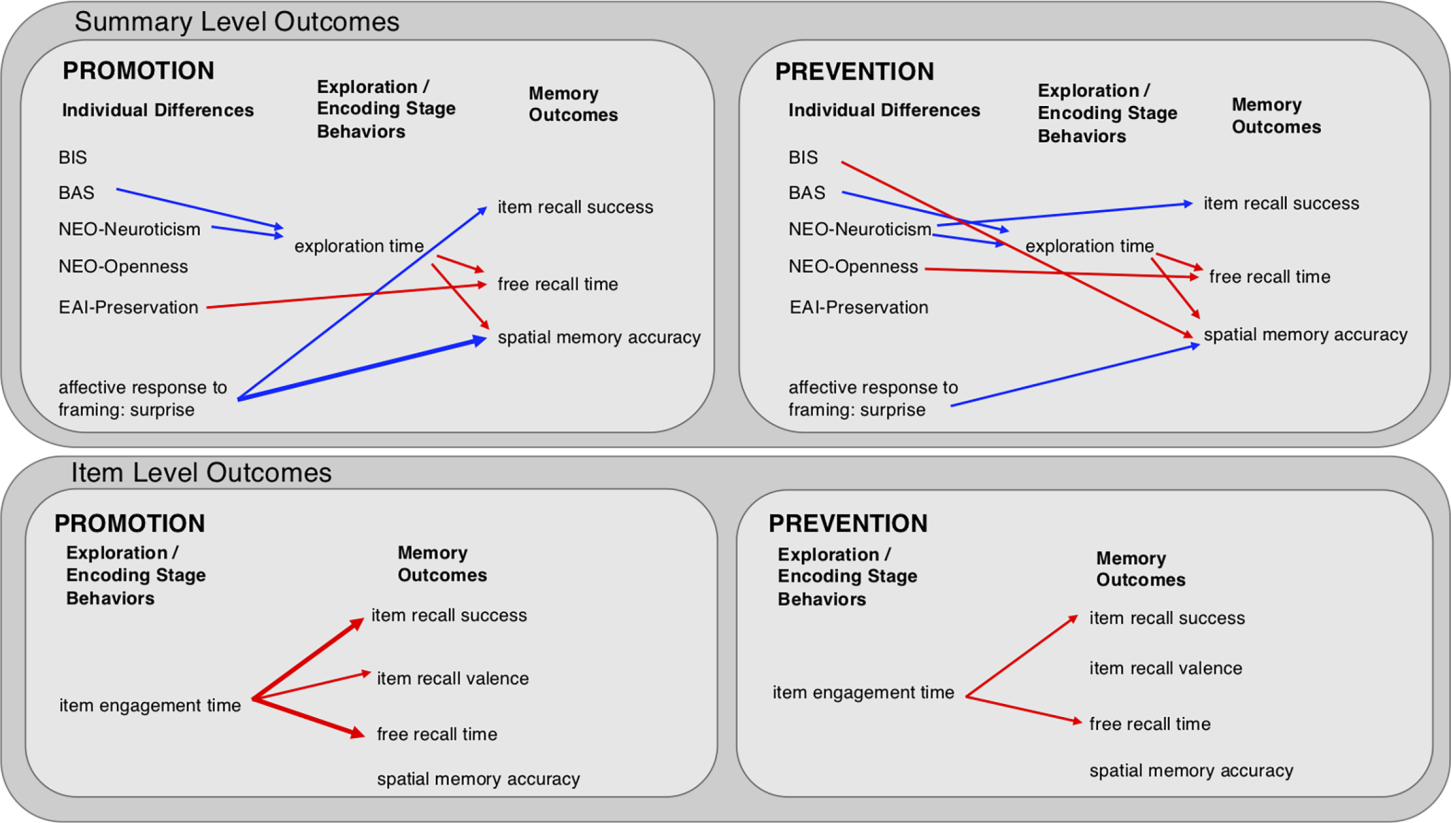
Key relationships between individual differences, exploration/encoding-stage behaviors, and memory outcomes. Relationships are shown separately for Promotion and Prevention framing conditions, for summary-level and item-level analysis outcomes. Only significant relationships are shown, in red for positive associations and blue for negative associations. Thick lines signify relationships that were significant in both framing conditions but stronger in one condition versus the other.

The prediction that participants would show greater exploration and correspondingly enhanced contextual memory in the Promotion vs. Prevention condition was not fulfilled in the present data, at least in terms of exploration time and measures of recall and spatial memory. Rather, we observed that exploration time and engagement during encoding were more tightly correlated to subsequent memory in the Promotion condition, suggesting that the Prevention manipulation disrupted typical depth-of-encoding relationships. Additionally, surprise expressed in response to the motivational manipulation was negatively associated with subsequent spatial memory, specifically in the Promotion condition. Finally, individual differences in personality and attitude variables predicted exploration and memory outcomes; regression analysis indicated both main effects of individual differences, and interactions with motivational context, on these outcome variables. These relationships, summarized in Fig 7, are examined in more detail below.

### Group- and item-level effects of motivational framing

Contrary to the hypothesis that exploration and memory would both be increased under Promotion vs. Prevention, and despite significant differences in facial affect expression during motivational induction between conditions, we did not find significant group-level effects of motivation condition, either on exploration time or on memory measures. Given the evidence that the groups’ overall motivation to explore the gallery was similar, we were able to further dissect relationships between motivation, spontaneous encoding behavior, and memory. We observed significant correlations between overall exploration and memory outcomes under Promotion, but not Prevention, conditions. These relationships at the summary level were corroborated by findings from item-level analyses: longer engagement with a given art item was associated with successfully recalling that item and describing the item for a longer period of time; and these relationships were significantly stronger under Promotion vs. Prevention. Taken together, these findings suggest that under a Promotion or reward-motivation context, memory outcomes are closely related to encoding behaviors, such as exploration. We expected this link between exploration and memory, given longstanding evidence that deeper encoding benefits memory performance [63]; the weakening of this relationship in the Prevention condition suggests that the Prevention manipulation disrupted typical depth-of-encoding mechanisms in memory formation.

Additionally, item-level analysis revealed that increasing item exploration time (engagement) was associated with the tendency to recall items more positively. This finding is in line with the well-established “mere exposure effect” [64], but this tendency was also stronger under Promotion vs. Prevention context. This is somewhat contradictory to prior literature suggesting that mere exposure effects might be amplified under negative affective contexts and reduced under positive affective contexts [65,66], but it is important to note that in these previous studies, participant stimulus exposure was tightly controlled and ability to volitionally engage or disengage was minimal. Under such circumstances, amplification of the mere exposure effect, and its association with negative affect, has been interpreted as an aversion to the new and unfamiliar [65]; given the volitional nature of exploration in the present study, it seems unlikely that aversion to novelty would support the mere exposure effect here. Arguably, our participants may have spent more time exploring art items that they subsequently recalled positively because they liked them more, and not vice versa (as the mere exposure effect would suggest); given the correlative nature of the observed relationship, we cannot determine its directionality. While this has yet to be clarified, at present these findings can be interpreted as additional evidence that under Promotion (vs. Prevention) context, memory outcomes were more closely related to encoding-stage behaviors.

These results also suggest that effects of motivational context may be relatively subtle when motivation is manipulated indirectly (as opposed to manipulation via the use of direct incentivization) and when behavior is characterized in naturalistic environments enabling relatively freeform action. Nevertheless, we observed a novel effect of motivational valence: exploration behavior and subsequent memory outcome appeared to be more tightly linked under Promotion vs. Prevention motivation. This stronger correlation suggests that exploratory or encoding-stage mechanisms might be relatively more important in a Promotion or reward-based motivational context; it is also possible that memories encoded during Prevention motivation may be constrained by consolidation or retrieval-stage mechanisms, limiting the impact of encoding behaviors. Much of the human research on motivated memory has investigated brain activation or behavior at the encoding stage, but recent work has demonstrated reward motivation effects on memory post-learning [67,68] as well as demonstrating effects of threat on retroactive memory consolidation [69]. At present, to our knowledge, no systematic comparison of the relative contributions of encoding vs. post-encoding processes to memory, as a function of motivational valence, exists. Such a comparison could potentially help inform the differential relationships between exploration and memory outcome observed as a function of valence in the present study.

### Stronger influence of surprise on memory in promotion condition parallels our previous arousal findings

Facial expressions during statement reading and subsequent behavior varied with framing condition. Participants expressed more surprise under Prevention than Promotion. Further, surprise was negatively associated with spatial memory in both conditions, but this relationship was stronger in the Promotion condition. Given that surprise is associated with heightened arousal, relative to a neutral emotion state [70], the inverse association between surprise and spatial memory can potentially be liked to prior findings from our laboratory [11], where high arousal predicted poorer spatial memory, specifically under reward. Effects of surprise on memory encoding have been mixed in the literature: surprising events can disrupt cognitive processing [71,72], but may also signify potential reward predictors during goal pursuit; enhanced memory has been observed for task-incidental, surprising stimuli encountered during reward anticipation [73]. In the present results, surprise appeared to have an impairing effect: memory for the exhibit space was impaired, and no enhancement in memory was observed for the exhibit statement itself, as a function of surprise. Only a minority of subjects mentioned the statement during recall (34 of 91 subjects with usable free recall data); but given that the exhibit statement was not obviously an art piece in the exhibit, it is possible that it was not considered test memoranda. A forced-response recognition memory paradigm, would have allowed direct evaluation of memory for the statement itself. Although memory for the surprising statement itself was not definitively assessed, these results add to a mixed literature regarding surprise effects on memory, indicating that surprise may disrupt memory for subsequent events.

### Individual differences and motivational framing interact to predict memory but not exploration time

Regression analyses examining the role of individual differences and their interactions with framing indicated both shared and distinct influences on behavior across motivational conditions. Importantly, individual differences in exploration time predicted hippocampally-dependent context memory measures (free recall time and spatial memory) but not item memory.

NEO-Neuroticism and BAS negatively predicted exploration time; these effects did not significantly interact with motivation condition. Less exploration with higher neuroticism was expected, given its associations with negative affect [74], increased volume in threat-related brain regions [75], and inhibitory effects of threat and anxiety on reward-seeking behavior [76]. In contrast, BAS as a negative predictor of exploration was unexpected, given prior associations between reward-seeking and exploratory behavior. However, BAS includes general tendencies towards goal pursuit [58] and, given experimental demand, this tendency could have led to more directed, rapid movement through the exhibit space, potentially reducing exploration time instead of increasing it. A significant negative correlation between BAS and item/wander time in the Promotion condition is in line with this interpretation, but given that BAS did not remain a significant predictor of item/wander time in regression analysis, this account remains tentative.

For all three measures of memory evaluated (item recall success, free recall time, and spatial memory accuracy), models examining the predictive role of trait individual differences were improved by the addition of a second step in the model, adding exploration time and interactions with framing as predictors. The significance of individual predictors, however, varied depending on the memory outcome. Neuroticism negatively predicted item memory: this is consistent with prior evidence linking high neuroticism to poorer semantic memory [77], possibly because of tendencies towards anxiety and decreased cognitive efficiency in highly neurotic individuals [78]. It is also notable that in these analyses, total exploration time was a significant predictor of free recall time and spatial memory, which are relatively dependent on hippocampus function, but not item memory, which is less reliant on the hippocampus. The results of these models thus suggest that hippocampally-dependent forms of memory might also be more closely related to exploration than item memory. This is consistent with prior evidence that exploration might promote memory via hippocampus-centric mechanisms [1].

Individual differences interacted with framing condition to predict time in free recall. This analysis was best fit as a two-step model, including both main effect and interaction terms. In this model, Exploration Time and Openness positively predicted free recall time across both Promotion and Prevention conditions; a significant Openness x Framing interaction further indicated that this relationship was stronger under Prevention. These results are consistent with prior research linking Openness to cognitive exploration and general mental ability [79,80] and DA system functioning [81]; additionally, as a proposed marker of resilience under adversity [82], Openness might especially benefit learning under Prevention framing.

Additionally, positive attitudes towards environmental preservation (as indexed by the EAI-Preservation subscale) predicted free recall time in the Promotion but not Prevention condition. While, to our knowledge, attitudes towards social issues have not previously been examined as predictors of regulatory fit, our findings are in line with prior research suggesting that framing manipulations can shape processing of environment- or sustainability-related information. Gain framing, compared to loss, has been associated with greater endorsement of climate change mitigation [55] and greater perceived environmental self-competence, engagement, and behavioral intention [83]. In contrast, loss framing has been linked to superior memory recall of climate change-related information [55], which authors interpreted as evidence of more analytical processing under negative affect. While this finding is inconsistent predictions of the present study, item and context memory were not separated, prohibiting direct comparison. Finally, the fact that free recall time scaled with EAI-Preservation is consistent with prior findings that people are more likely to engage with learned environmental information from a trusted source [53]; high EAI-Preservation individuals may have been more likely to trust the information in our experiment (ostensibly presented by Duke University’s Nicholas School of the Environment) and thus more inclined to engage with and encode that information. Thus, our EAI-Preservation x Framing interaction dovetails with prior findings in the environmental communications literature, but also suggests more broadly that communication outcomes might depend on the way that information is provided and memory is assessed. In contrast to item memory for facts (which might benefit from loss framing, as suggested by [55]), our results suggest that memory for more elaborative or complex environmental information may benefit from Promotion motivation or gain framing, especially if individuals are positively inclined towards environmentalism to begin with. Finally, spatial memory was positively predicted by both Exploration Time and BIS, qualified by a significant BIS x Framing interaction indicating that BIS effects were stronger in the Prevention condition. This significant interaction might reflect a regulatory fit effect, consistent with prior evidence of enhanced cognitive performance under state-trait congruency [51].

Importantly, Neuroticism and BIS had differing effects on memory. Despite their conceptual overlap as measures of negative affect and punishment sensitivity, Neuroticism was inversely associated with item memory, while BIS was positively associated with spatial memory, particularly in the Prevention condition. While both Neuroticism and BIS have been associated with negative affect, the constructs are distinct [84]. As a tendency towards goal pursuit, BIS may have promoted goal-relevant processing and enhanced exhibit memory, particularly under Prevention framing: i.e., reflecting a regulatory fit effect. In contrast, Neuroticism might have been associated with goal-irrelevant negative affect and memory impairment. Such differences would be consistent with data indicating the importance of goal relevance in determining the influence of affect on memory outcomes [85].

(Note: To investigate for potential affective mechanisms underlying the diverging influences of Neuroticism and BIS on memory, we conducted exploratory analyses relating individual differences in these traits to expressed surprise during statement reading. Neuroticism and expressed surprise were positively associated under Promotion (no relationship under Prevention); given the inverse relationship between surprise and subsequent spatial memory, these findings support the idea that high Neuroticism could have led to goal-irrelevant negative affect, expressed as surprise, that disrupted memory. In contrast, relationships between BIS and expressed surprise were negative in both conditions. These results, shown in S1 Fig in the Supporting Information, did not reach statistical significance so interpretation remains speculative on our part, but hint at relationships between trait measures, affect, and cognitive outcomes to be explored further in future research.)

### Implications for environmental communication

The present study offered a unique opportunity to characterize motivated engagement with and memory for sustainability-relevant information, with important implications for the environmental communications literature. Climate change and environmental crisis are important but complex, highly uncertain issues: communicating relevant information accurately and in a way that encourages prosocial behavior is an important public concern. Many studies examining framing effects in the environmental communications literature use reported attitudes as outcome, while a more limited number have examined cognitive outcomes such as memory for environmental information [55]. Our results demonstrate that individual differences and motivational context can influence how people engage with and remember environmental information. Further, our results suggest that these factors might differentially influence item and context memory. To our knowledge, memory for item versus context information has not been clearly differentiated in the communications literature (environmental communication, health communication, or otherwise). Given cognitive neuroscience evidence of hippocampal involvement in concept learning and decision-making [86–88], it may be useful to compare whether promoting hippocampally-dependent context memory (as opposed to item memory) for information could lead to improved decision outcomes in applied communications settings. It is possible that distinguishing between item and contextual memory may refine and improve applied communications efforts and dissemination of information, advancing public understanding of complex issues such as sustainability science.

### Experimental limitations, unresolved questions, and new hypotheses

The present study sought to characterize relationships between motivation, exploration, and memory in a complex, real-life setting that demanded more consideration of individual differences, but they were not the primary focus of the study. Our study sample is adequate for our primary hypotheses, but follow-up work will enable evaluation of the replicability and generalizability of our individual difference results.

Our data reveal an inverse relationship between surprise and spatial memory, which was stronger under Promotion. While this relationship is consistent with our prior work demonstrating an inverse correlation between arousal and spatial memory [11], it is important to note that no ongoing measure of physiological arousal was collected in the present study. Follow-up studies could confirm this interpretation using online measures of arousal–for example, ambulatory monitors to track heart rate [89], and mobile eyetracking to index pupil dilation [90,91], as potential measures of physiological arousal during exploration.

While we were able to characterize motivated exploration and memory behaviors in a real-life spatial context, some environmental constraints limited our data analyses. For example, while it would have been interesting to examine the rate at which participants approached and withdrew from art items, participants often withdrew from one item and approached another in a single movement. However, given that other studies have meaningfully characterized human locomotion in relation to exploration and dopaminergic function in a real-life environment [30,92], future studies would benefit from use of a stimulus environment that enables more nuanced characterization of such approach and withdrawal behaviors.

Follow-up investigations could incorporate additional outcome measures that would help clarify observations from the present data. First, engagement with the individual art items was associated with subsequent emotional valence in recall. However, no direct ratings of each art item were solicited from participants. Second, our study design evaluated memory at a 24-hour interval; immediate memory was not assessed to avoid influencing the visit duration. Thus we were not able to distinguish attentional mechanisms at encoding from post-encoding consolidation processes [67,93,94]. Given that our data suggest a tighter link between encoding-stage exploration behaviors and subsequent memory in the Promotion condition, examining both immediate and delayed memory could help disentangle relative contributions of encoding versus post-encoding mechanisms to memory performance as a function of motivational context.

Finally, it is important to note that an extensive literature has characterized sex differences in spatial navigation and memory performance, with males generally outperforming females on wayfinding and spatial memory tasks [95–98], potentially due to greater acute stress responses during spatial task performance in females [99]. Analyses examining our outcome measures as a main effect of gender are available in S5 Table in the Supporting Information; no significant differences were observed. Given the present study’s focus and the lack of a significant main effect of gender on any of our outcome measures, we elected not to conduct an in-depth examination of the potential influence of gender on performance. However, this remains an important direction to be fully investigated in future research.

Our findings generate exciting new hypotheses to be explored in future work. Notably, the observation that the relationship between exploration behavior at encoding and subsequent memory outcome was disrupted under Prevention framing suggests that encoding-stage mechanisms would be relatively more important under Promotion or appetitive motivation, while memory under Prevention or avoidance motivation contexts would depend relatively more on post-encoding consolidation or retrieval-stage mechanisms. Our results also suggest that motivational state-trait congruency might facilitate memory formation. While similar results have been demonstrated in an academic setting using a regulatory fit manipulation [51], potential interactions of state and trait variables have not been well-characterized in the motivated memory literature, and the neural mechanisms underlying such effects remain to be delineated.

### Conclusions

The present study provided a novel investigation into motivated exploration and memory for a real-life, naturalistic environment. We observed that motivational framing did not affect overall motivation to remain in the novel spatial context, but instead altered the relationship between encoding behavior and memory outcomes. Although increased exploratory behavior is one mechanism of improved subsequent memory performance linked to hippocampal function [1,3,6], the current findings suggest that motivational contexts elicit mechanisms that constrain memory performance independently of effects on exploration or encoding, at least in terms of exploration time. Additionally, individual differences in personality, attitudes, and affective response interacted with motivational context to improve predictions of behavior. Given our stimuli, these findings also help characterize predictors of motivated engagement with and memory for sustainability-related information. By providing a characterization of multiple, interactive influences on memory in a naturalistic environment, the present data offer additional insights into mechanisms for further investigation and an account that more closely parallels how motivated memory unfolds during daily life in a complex world.

## Supporting information

S1 Text Supplementary methods: Facial expression analysis. (PDF)

S2 Text Supplementary results. Exploration and Memory Measures as a Main Effect of Group.
(PDF)

S1 Table Data present for each experimental measure. (PDF)

S2 Table Demographic, personality, and environmental attitude variables of the sample, separated by framing condition. Means are presented with standard deviations in brackets. Usable N for each data measure present noted.
(PDF)

S3 Table Correlational relationships between individual difference measures and dependent measures of exploration and memory behavior collapsed across Promotion and Prevention framing conditions (text with white background); in the Promotion condition (text with red background); and in the Prevention condition (text with blue background). (Pearson correlation coefficients, significance uncorrected for multiple comparisons.) ^*p* < .10; *p < .05; **p < .01.
(PDF)

S4 Table Hierarchical multiple regression model with item/wander time proportion as a DV and individual differences and framing condition as predictors (Indiv x Framing interaction terms entered at Step 2). ^*p* < .10; *p < .05; **p < .01.
(PDF)

S5 Table Exploration time, number of items recalled, item valence in free recall, free recall time, and spatial memory performance, separated by gender. (DOCX)

S1 Fig Given that Neuroticism was associated with impaired memory while BIS was associated with enhanced memory, we plotted relationships between (a) NEO-Neuroticism and expressed surprise during statement reading; (b) BIS and expressed surprise during statement reading; to investigate for affective mechanisms underlying these diverging effects. A positive relationship between increasing Neuroticism and surprise in the Promotion condition suggests that highly Neuroticism individuals expressed more surprise (and may have experienced increased emotional arousal), leading to impaired subsequent exhibit memory. In contrast, increasing BIS was associated with decreasing surprise in both the Promotion and Prevention conditions, with a more robust association under Prevention. These results suggest that Neuroticism, but not BIS, was positively associated with emotional arousal and potentially, task-irrelevant negative affect: leading to memory impairment with increasing Neuroticism but not BIS. Further, these relationships interacted with motivational context. These results are speculative, given that none of the analyses reached statistical significance, but hint at potential mechanisms to be explored more fully in future research.
(TIFF)
