# Supplementary material for: Motivational valence alters memory formation without altering exploration of a real-life spatial environment
Source: PLoS One. 2018 Mar 20;13(3):e0193506. doi: 10.1371/journal.pone.0193506 (PMC5860699; doi:10.1371/journal.pone.0193506)
Supplement: S1 Text — (PDF) [file pone.0193506.s001.pdf]

S1 Text

Classification of facial expressions in the present study followed the state-of-the-art algorithm proposed in (Hashemi, Qiu, & Sapiro, 2015). The proposed algorithm provides a fully automatic, robust, and pose-invariant method for estimating facial expressions in images by first detecting and extracting image descriptors (local binary patterns) around specific regions of the face (i.e., eyes, eyebrows, nose, and mouth; 19 landmarks in total), and then training a pose-invariant facial expression classifier using Support Vector Machines. The developed algorithm achieved high agreement across multiple facial expression datasets including CK+, BU3D-FE, and Multi-Pie (Gross, Matthews, Cohn, Kanade, & Baker, 2008; Kanade, Cohn, & Tian, 2000; Lucey et al., 2010; Yin, Wei, Wang, & Rosato, 2006). The algorithm also has been clinically validated in pediatric populations (Hashemi, Campbell, et al., 2015). For the present study, angry, happy, sad, surprised, or neutral facial expression were considered.

S1 Text References

Gross, R., Matthews, I., Cohn, J. F., Kanade, T., & Baker, S. (2008). *Multi-PIE.* Paper presented at the IEEE International Conference on Automatic Face and Gesture Recognition, Amsterdam, Netherlands.

Hashemi, J., Campbell, K., Carpenter, K., Harris, A., Qiu, Q., Tepper, M., . . . Sapiro, G. (2015). *A scalable app for measuring autism risk behaviors in young children: a technical validity and feasibility study.* Paper presented at the EAI International Conference on Wireless Mobile Communication and Healthcare, London, UK.

Hashemi, J., Qiu, Q., & Sapiro, G. (2015). *Cross-modality pose-invariant facial expression.* Paper presented at the IEEE International Conference on Image Processing, Quebec City, Canada.

Kanade, T., Cohn, J. F., & Tian, Y. (2000). *Comprehensive database for facial expression analysis.* Paper presented at the Fourth IEEE International Conference on Automatic Face and Gesture Recognition, Grenoble, France.

Lucey, P., Cohn, J. F., Kanade, T., Saragih, J., Ambadar, Z., & Matthews, I. (2010). *The extended Cohn-Kanade dataset (CK+): a complete expression dataset for action unit and emotion-specified expression.* Paper presented at the Third International Workshop on CVPR for Human Communicative Behavior Analysis, San Francisco, USA.

Yin, L., Wei, X., Wang, J., & Rosato, M. (2006). *A 3D facial expression database for facial behavior research.* Paper presented at the IEEE International Conference on Automatic Face and Gesture Recognition, Southhampton, UK.
