# Supplementary material for: Motivational valence alters memory formation without altering exploration of a real-life spatial environment
Source: PLoS One. 2018 Mar 20;13(3):e0193506. doi: 10.1371/journal.pone.0193506 (PMC5860699; doi:10.1371/journal.pone.0193506)
Supplement: S2 Text — Exploration and Memory Measures as a Main Effect of Group. (PDF) [file pone.0193506.s002.pdf]

S2 Text

Supplementary Results: Exploration and Memory Measures as a Main Effect of Group

Exploration Time. Exploration time in the exhibit (entry to exit) was calculated from video footage. Exploration time averaged ~20 minutes in each condition and, contrary to prediction, did not significantly differ across framing condition [$t(93) = -.114$, $p = .909$, Cohen's $d = -.023$], with a large amount of variability [in Promotion: $M(49) = 1185.6$ seconds, $SD = 796.9$ seconds; in Prevention: $M(46) = 1204.5$ seconds, $SD = 824.2$ seconds].

Item/Wander Time. The relative proportion of total exhibit exploration time that was spent engaging with specific art items was calculated from video footage. On average, participants in both framing conditions spent most of their total exhibit exploration time engaging with specific art items [proportion scores in Promotion: $M(43) = .8355$, $SD = .087$; in Prevention: $M(39) = .824$, $SD = .139$]. These proportions did not significantly differ across conditions [$t(80) = .474$, $p = .637$, Cohen's $d = .104$].

Free Recall: Number of Items Recalled. The exhibit contained eight art objects. Free recall transcripts (N=91) were scored for these eight objects, as well as four additional objects spontaneously mentioned (exhibit statement, entry decoration, exhibit program, and decorative tiles on the floor), for a maximum of twelve items recalled. On average, participants recalled approximately six items [Promotion: $M(50) = 6.38$, $SD = 2.49$; Prevention: $M(41) = 5.95$, $SD = 3.04$]. Number of items recalled did not significantly differ across conditions [$t(89) = .740$, $p = .461$, Cohen's $d = .156$].

Free Recall: Valence of Items Recalled. We examined whether item valence in free recall (whether items were recalled as emotionally positive, negative, neutral or ambivalent) differed with motivational context. Eighty-eight subjects were included in this analysis (three

participants of N=91 did not name any specific items). Items mentioned during free recall were coded by two independent, condition-blind raters for valence (positive, negative, neutral/not-stated, or ambivalent). Inter-rater reliability was good (72.8% overlap; Cohen's kappa = .55) (Fleiss, 1981) across 9 items (the 8 art pieces, plus exhibit statement). Fewer than 15 participants mentioned entry decoration, exhibit program and floor tiles; these three items were excluded from valence analysis. Reports with inconsistent ratings, where one rating was neutral/not-stated and the other rating was valenced (positive, negative, or ambivalent) were classified as the valenced rating (20.2% of trials). Items with mismatched valence ratings were eliminated from analysis (6.6% of trials).

Participants generally recalled art exhibit items as positively or neutrally valenced: across the sample, objects were rated as positive (52.6%), neutral (32.5%), negative (11.7%), or ambivalent (3.2%). To examine whether proportions of emotional valences expressed in free recall significantly differed as a function of framing condition, a mixed-effects logistic regression was implemented (following the analytic procedure used to examine facial expressions during statement reading across framing groups; refer to Main Text). Framing condition was defined as a fixed effect and subject and item were defined as random effects, permitting us to examine valence of each item recalled as a categorical outcome, nested within-subject, nested within experimental group. Models were calculated as binomial contrasts between outcome categories (Dobson & Barnett, 2008). None of these contrasts reached significance, indicating that item valence in free recall did not significantly differ as a function of motivational context.

<u>Free Recall: Memory Elaboration (Time in Free Recall).</u> The length of time that each participant spent in free recall was calculated from the audio recording and treated as an index of memory elaboration. Given the relatively open-ended instructions that participants were provided, memory elaboration (total recall time) was highly variable.

Total recall time averaged ~4.4 minutes across framing conditions [Promotion: $M(50)$ = 261.62 seconds, SD = 231.75; Prevention: $M(41)$ = 265.63 seconds, SD = 191.93]. Recall time did not significantly differ as a function of condition [$t(89) = -.089$, $p = .930$, Cohen's $d = .019$].

Spatial Memory Test. The spatial memory test assessed location memory for the eight art items (N=93). Accuracy was scored as a proportion of 8 (correct/incorrect for each item). Accuracy was high across both groups [Promotion: $M(50)$ = 0.770, SD = .278; Prevention: $M(43)$ = 0.799, SD = .311] and did not significantly differ with framing [$t(76) = -.125$, $p = .901$, Cohen's $d = -.054$]. Memory confidence, rated for each item on a Likert scale from 1 (guessing) to 5 (very confident), was relatively high [Promotion: $M(50)$ = 4.12, SD = .951; Prevention: $M(43)$ = 4.20, SD = 1.05], was positively correlated with memory accuracy [$r(93) = .442$, $p < .001$], and did not significantly differ across conditions [$t(91) = -.381$, $p = .704$, Cohen's $d = -.079$].

Supplementary Results: Trait Individual Differences as Predictors of Item/Wander Time

N=71 (N=38 Promotion, N=33 Prevention) were usable in this analysis (shown in S3 Table).

S2 Text References

Dobson, A. J., & Barnett, A. (2008). *An introduction to generalized linear models*. Boca Raton: CRC Press.

Fleiss, J. L. (1981). *Statistical methods for rates and proportions (second edition)*. New York: John Wiley.
