# Supplementary material for: Motivational valence alters memory formation without altering exploration of a real-life spatial environment
Source: PLoS One. 2018 Mar 20;13(3):e0193506. doi: 10.1371/journal.pone.0193506 (PMC5860699; doi:10.1371/journal.pone.0193506)
Supplement: S1 Table — (PDF) [file pone.0193506.s003.pdf]

S1 Table

Variable	Number of subjects
Total number of subjects	98 (Promotion = 52, Prevention = 46)
Free recall data	91 (Promotion = 50, Prevention = 41)
Spatial task data	93 (Promotion = 50, Prevention = 43)
BIS/BAS	88 (Promotion = 47, Prevention = 41)
NEO-FFI	91 (Promotion = 47, Prevention = 44)
EAI	96 (Promotion = 52, Prevention = 44)
GoPro (facial expression) video	83 (Promotion = 43, Prevention = 40)
Lorex (overhead) video	82 (Promotion = 43, Prevention = 39)
