# Supplementary material for: Motivational valence alters memory formation without altering exploration of a real-life spatial environment
Source: PLoS One. 2018 Mar 20;13(3):e0193506. doi: 10.1371/journal.pone.0193506 (PMC5860699; doi:10.1371/journal.pone.0193506)
Supplement: S2 Table — Means are presented with standard deviations in brackets. Usable N for each data measure present noted. (PDF) [file pone.0193506.s004.pdf]

S2 Table

	**Promotion (N=52)**	**Prevention (N=46)**	**Group difference test and effect size**
Age (years)	N=50<br>32.94 (15.34)	N=39<br>32.82 (12.12)	t(87) = .040,<br>p = .968, Cohen's d = .009
Mean education level	N=46<br><br>Some highschool (8)<br>Highschool (8)<br>Some college (13)<br>College (6)<br>Some postgrad (5)<br>Postgrad (6)	N=37<br><br>Some highschool (6)<br>Highschool (8)<br>Some college (6)<br>College (5)<br>Some post grad (8)<br>Postgrad (4)	X^2 = 3.109, df = 5, p = .683,<br>φ = .194
Gender	25 male<br>27 female	22 male<br>24 female	X^2 = .001, df = 1, p = .980,<br>φ = .003
BAS-Drive	N=47<br>11.66 (2.13)	N=42<br>12.00 (2.62)	t(87) = -.675, p = .501,<br>Cohen's d = .142
BAS-Fun Seeking	N=47<br>12.04 (2.21)	N=42<br>11.93 (2.83)	t(87) = .213, p = .832,<br>Cohen's d = .043
BAS-Reward Responsivity	N=47<br>17.87 (1.93)	N=42<br>18.02 (1.63)	t(87) = -.397, p = .692,<br>Cohen's d = .084
BIS	N=47<br>20.87 (3.17)	N=42<br>19.76 (3.50)	t(87) = 1.569, p = .120,<br>Cohen's d = .084
NEO-FFI Agreeableness	N=47<br>37.45 (6.73)	N=44<br>38.70 (6.65)	t(89) = -.897, p = .372,<br>Cohen's d = .187
NEO-FFI Conscientiousness	N=47<br>41.47 (10.01)	N=44<br>40.73 (9.97)	t(89) = .353, p = .725,<br>Cohen's d = .074
NEO-FFI Extroversion	N=47<br>36.79 (8.31)	N=44<br>36.84 (8.22)	t(89) = -.031, p = .975,<br>Cohen's d =
NEO-FFI Neuroticism	N=47<br>27.40 (9.79)	N=44<br>25.89 (9.97)	t(89) = .733, p = .466,<br>Cohen's d = .006
NEO-FFI Openness to Experience	N=47<br>40.51 (7.56)	N=44<br>38.11 (8.05)	t(89) = 1.465, p = .147,<br>Cohen's d = .307
EAI-24 Preservation	N=52<br>4.91 (0.97)	N=44<br>5.18 (0.82)	t(94) = -1.470, p = .145,<br>Cohen's d = .301
