# Supplementary material for: Motivational valence alters memory formation without altering exploration of a real-life spatial environment
Source: PLoS One. 2018 Mar 20;13(3):e0193506. doi: 10.1371/journal.pone.0193506 (PMC5860699; doi:10.1371/journal.pone.0193506)
Supplement: S3 Table — (Pearson correlation coefficients, significance uncorrected for multiple comparisons.) ^p < .10; *p < .05; **p < .01. (PDF) [file pone.0193506.s005.pdf]

S3 Table

	Exploration Time	Item/Wander Time	Number of Items Recalled	Free Recall Time	Spatial Memory Accuracy
BAS-Drive	r(86) = -.241*	r(73) = -.137	r(81) = .029	r(81) = -.118	r(83) = -.006
	r(45) = -.337*	r(38) = -.330*	r(45) = -.056	r(45) = -.226	r(45) = -.263^
	r(41) = -.172	r(35) = -.034	r(36) = .111	r(36) = .009	r(38) = .198
BAS-Fun Seeking	r(86) = -.198^	r(73) = -.098	r(81) = .093	r(81) = -.008	r(83) = .064
	r(45) = -.144	r(38) = -.256	r(45) = .092	r(45) = -.098	r(45) = -.108
	r(41) = -.243	r(35) = -.001	r(36) = .083	r(36) = .099	r(38) = .218
BAS-Reward Responsivity	r(86) = -.080	r(73) = .019	r(81) = .005	r(81) = -.028	r(83) = -.023
	r(45) = -.027	r(38) = -.138	r(45) = .217	r(45) = .116	r(45) = -.054
	r(41) = -.150	r(35) = .150	r(36) = -.235	r(36) = -.278	r(38) = .013
BIS	r(86) = -.116	r(73) = -.186	r(81) = .052	r(81) = .018	r(83) = .197^
	r(45) = -.145	r(38) = -.073	r(45) = .049	r(45) = -.035	r(45) = -.049
	r(41) = -.075	r(35) = -.277	r(36) = .010	r(36) = .092	r(38) = .446**
NEO-FFI Agreeableness	r(89) = .065	r(75) = -.163	r(84) = .009	r(84) = .056	r(86) = .027
	r(45) = .214	r(38) = -.124	r(45) = .130	r(45) = .100	r(45) = .082
	r(44) = -.089	r(37) = -.193	r(39) = -.082	r(39) = -.004	r(41) = -.038
NEO-FFI Conscientiousness	r(89) = .016	r(75) = .072	r(84) = -.032	r(84) = -.068	r(86) = .046
	r(45) = .122	r(38) = -.110	r(45) = .126	r(45) = -.023	r(45) = .104
	r(44) = -.087	r(37) = .199	r(39) = -.188	r(39) = -.132	r(41) = -.006
NEO-FFI Extroversion	r(89) = -.069	r(75) = -.176	r(84) = .065	r(84) = -.052	r(86) = .049
	r(45) = .059	r(38) = -.178	r(45) = .081	r(45) = -.167	r(45) = .020
	r(44) = -.190	r(37) = -.180	r(39) = .054	r(39) = .127	r(41) = .078
NEO-FFI Neuroticism	r(89) = -.255*	r(75) = -.177	r(84) = -.103	r(84) = -.174	r(86) = -.078
	r(45) = -.248	r(38) = -.032	r(45) = .078	r(45) = -.220	r(45) = -.132
	r(44) = -.258^	r(37) = -.291^	r(39) = -.284^	r(39) = -.120	r(41) = -.023
NEO-FFI Openness to Experience	r(89) = .035	r(75) = -.076	r(84) =.233*	r(84) = .184^	r(86) = .154
	r(45) = .263^	r(38) = -.059	r(45) = .351*	r(45) = .066	r(45) = .175
	r(44) = -.168	r(37) = -.096	r(39) = .095	r(39) = .348*	r(41) = .153
EAI-Preservation	r(93) = .081	r(80) = -.024	r(83) = .102	r(91) = .192^	r(92) = .233*
	r(49) = .236	r(43) = -.139	r(46) = .174	r(50) = .302*	r(50) = .224
	r(44) = -.111	r(37) = .081	r(37) = .042	r(41) = -.020	r(42) = .244
