# Supplementary material for: Motivational valence alters memory formation without altering exploration of a real-life spatial environment
Source: PLoS One. 2018 Mar 20;13(3):e0193506. doi: 10.1371/journal.pone.0193506 (PMC5860699; doi:10.1371/journal.pone.0193506)
Supplement: S4 Table — ^p < .10; *p < .05; **p < .01. (PDF) [file pone.0193506.s006.pdf]

S4 Table

**MODEL 1<br>(R^2 = .057, F(6,64) = .647, p = .692)**			
**Variable**	**β Coefficient**	**t value**	**p value**
Promotion/Prevention Framing	.072	.563	.576
BAS (Composite)	-.074	-.571	.570
BIS	-.120	-.860	.393
NEO Neuroticism	-.156	-1.039	.303
NEO Openness to Experience	.057	.400	.690
EAI Preservation	-.019	-.143	.887
**MODEL 2<br>(R^2 = .132, F(11,59) = .814, p = .626)**			
**Variable**	**β Coefficient**	**t value**	**p value**
Promotion/Prevention Framing	.094	.700	.486
BAS (Composite)	.103	.542	.590
BIS	-.234	-1.051	.298
NEO Neuroticism	-.361	-1.418	.161
NEO Openness to Experience	.156	.690	.493
EAI Preservation	.051	.214	.831
BAS (Composite) x Framing	-.234	-1.299	.199
BIS x Framing	.102	.507	.614
NEO Neuroticism x Framing	.257	1.099	.276
NEO Openness to Experience x Framing	-.125	-.615	.541
EAI Preservation x Framing*	-.089	-.403	.688
