# Supplementary material for: Motivational valence alters memory formation without altering exploration of a real-life spatial environment
Source: PLoS One. 2018 Mar 20;13(3):e0193506. doi: 10.1371/journal.pone.0193506 (PMC5860699; doi:10.1371/journal.pone.0193506)
Supplement: S5 Table — (DOCX) [file pone.0193506.s007.docx]

*S5 Table.* Exploration time, number of items recalled, item valence in free recall, free recall time, and spatial memory performance, separated by gender.

|  | | **Males (N=47)** | | **Females (N=51)** | | **Group Difference Test** | | |
| --- | --- | --- | --- | --- | --- | --- | --- | --- |
|  | | **usable n** | **mean (SD)** | **usable n** | **mean (SD)** | **t-statistic** | **p-value** | **Cohen’s d** |
| **Exploration time (seconds)** | | 47 | 1283 (685.0) | 48 | 1107.6 (907.8) | 1.066 | .289 | .219 |
| **Item/wander time (proportion of total exploration time spent in item engagement)** | | 38 | 0.837 (.092) | 44 | 0.824 (.131) | .511 | .611 | .114 |
| **Number of items recalled** | | 45 | 6.16 (2.61) | 46 | 6.22 (2.90) | -.107 | .915 | -.002 |
| **Item valence in free recall (percentages)** | **Positive** | 44 | 49.0 (29.9) | 44 | 56.8 (27.0) | -1.282 | .203 | -.274 |
|  | **Negative** | 44 | 13.9 (17.3) | 44 | 9.9 (14.0) | 1.211 | .229 | .254 |
|  | **Neutral** | 44 | 35.2 (32.7) | 44 | 30.2 (31.0) | .738 | .463 | .157 |
|  | **Ambivalent** | 44 | 2.4 (5.9) | 44 | 4.5 (11.7) | -1.018 | .311 | -.227 |
| **Time in free recall (seconds)** | | 45 | 276.11 (207.39) | 46 | 251.02 (221.04) | .558 | .578 | .117 |
| **Spatial memory performance (proportion accuracy)** | | 45 | 0.756 (.319) | 46 | 0.810 (.267) | -.894 | .374 | -.184 |
| **Spatial memory confidence (5-point Likert scale, from 1= “guessing” to 5=”very confident”)** | | 45 | 4.21 (.924) | 48 | 4.10 (1.06) | .521 | .603 | .111 |
