# Supplementary figures and images for: Motivational valence alters memory formation without altering exploration of a real-life spatial environment
Source: PLoS One. 2018 Mar 20;13(3):e0193506. doi: 10.1371/journal.pone.0193506 (PMC5860699; doi:10.1371/journal.pone.0193506)

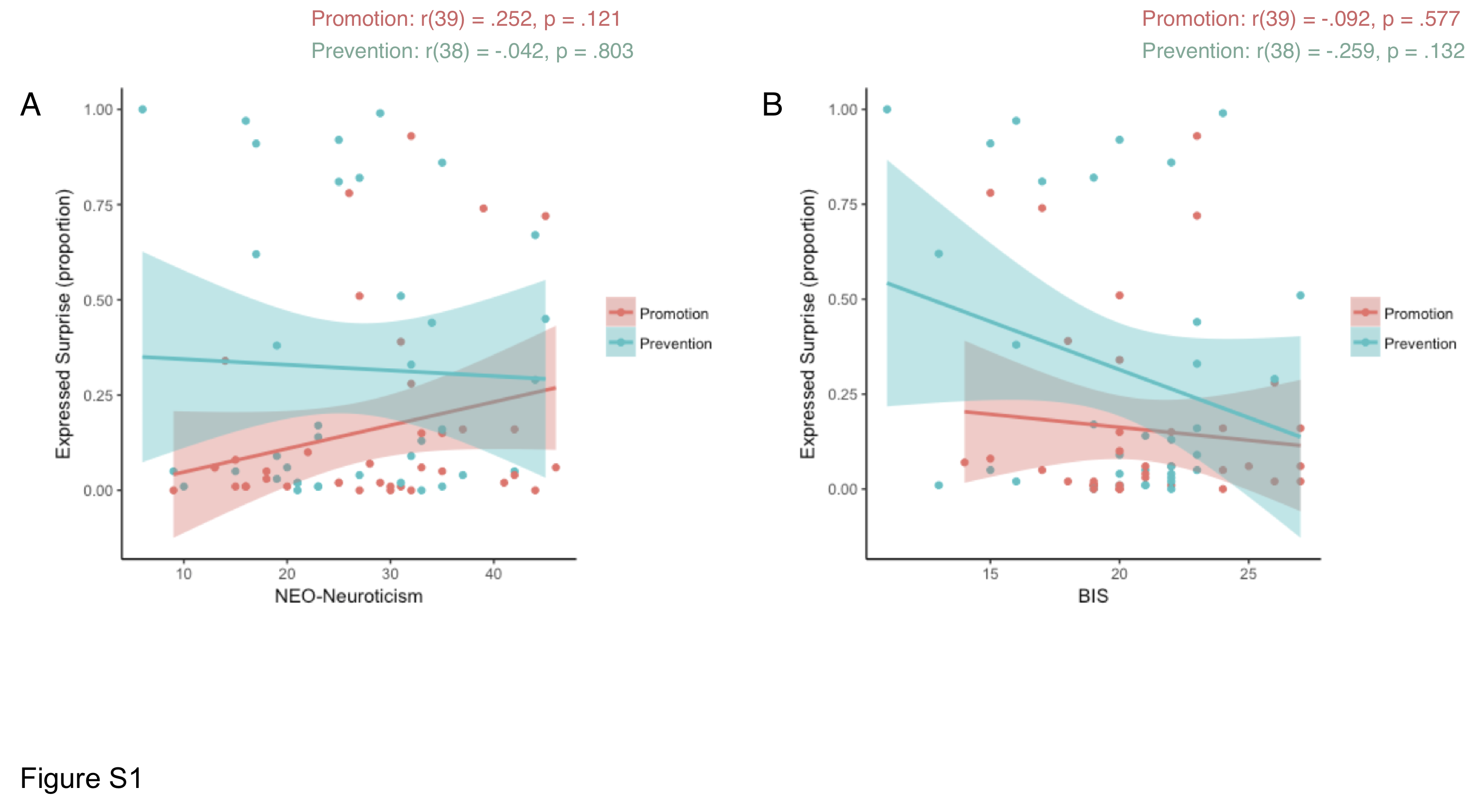

Supplement: S1 Fig — Given that Neuroticism was associated with impaired memory while BIS was associated with enhanced memory, we plotted relationships between (a) NEO-Neuroticism and expressed surprise during statement reading; (b) BIS and expressed surprise during statement reading; to investigate for affective mechanisms underlying these diverging effects. A positive relationship between increasing Neuroticism and surprise in the Promotion condition suggests that highly Neuroticism individuals expressed more surprise (and may have experienced increased emotional arousal), leading to impaired subsequent exhibit memory. In contrast, increasing BIS was associated with decreasing surprise in both the Promotion and Prevention conditions, with a more robust association under Prevention. These results suggest that Neuroticism, but not BIS, was positively associated with emotional arousal and potentially, task-irrelevant negative affect: leading to memory impairment with increasing Neuroticism but not BIS. Further, these relationships interacted with motivational context. These results are speculative, given that none of the analyses reached statistical significance, but hint at potential mechanisms to be explored more fully in future research. (TIFF) [file pone.0193506.s008.tiff]
